# Identification and Analysis of InDel Variants in Key Hippo Pathway Genes and Their Association with Growth Traits in Four Chinese Sheep Breeds

**DOI:** 10.3390/vetsci12030283

**Published:** 2025-03-18

**Authors:** Beibei Zhang, Wanxia Zhao, Xiaoqin Tang, Meng Zhou, Yanbo Qiu, Shuhui Wang, Xiuzhu Sun

**Affiliations:** 1 College of Grassland Agriculture, Northwest A&F University, Yangling 712100, China; double08008@nwafu.edu.cn (B.Z.); 2022056888@nwafu.edu.cn (M.Z.); qiuyanbo1999@nwafu.edu.cn (Y.Q.); 2 College of Animal Science and Technology, Northwest A&F University, Yangling 712100, China; wanxia@nwafu.edu.cn (W.Z.); txq@nwafu.edu.cn (X.T.)

**Keywords:** Hippo signaling pathway, InDel, sheep breeding, growth traits

## Abstract

This study aimed to identify insertion–deletion (InDel) variants in key genes of the Hippo signaling pathway in four Chinese sheep breeds: Tong sheep (TS), Hu sheep (HS), Small Tail Han sheep (STHS), and Lanzhou large-tailed sheep (LLTS). InDel variants in *MST1/2*, *LATS1/2*, *SAV1*, *MOB1A/B*, and *YAP/TAZ* genes were screened using public databases and identified through PCR amplification, gel electrophoresis, and sequencing. This study identified significant associations between InDel variants and growth traits across the four breeds. Specifically, three loci in the *MST1* gene were significantly associated with chest circumference, body height, and body weight in STHS, HS, and TS. Four loci in *MST2* influenced hip height, body weight, and chest circumference across all breeds (*p* < 0.05). Additionally, two loci in *YAP* significantly affected body height, chest circumference, and body length in LLTS, STHS, and TS, while three loci in the *MOB1A* gene strongly impacted cannon circumference in all breeds (*p* < 0.05). These InDel variants may serve as potential molecular markers for breeding. These findings highlight the potential of these InDel variants as molecular markers for sheep breeding and provide valuable resources for improving growth traits in sheep through molecular breeding.

## Simple Summary

The Hippo signaling pathway affects the growth and development of animals; however, few studies have focused on the InDels of key genes in the sheep Hippo signaling pathway and their effects on growth traits. In this study, InDel variants of key genes in the Hippo signaling pathway were identified in four Chinese sheep breeds. A total of 23 InDel sites associated with the *MST1*, *MST2*, *YAP*, *TAZ*, and *MOB1A* genes were screened, and 12 of them were found to be significantly associated with growth traits. These sites could serve as important molecular markers influencing the growth and development of sheep. Our study provides valuable resources for molecular breeding in sheep.

## 1. Introduction

Sheep (Ovis aries) have been a significant economic livestock species since the Neolithic Age, providing meat, wool, skin, and milk to humans [1]. As a result, the income of farmers or breeders is heavily influenced by the size and weight of their animals. Therefore, growth-related traits in livestock, particularly in sheep, such as body weight (BW) and body size, play a critical role in enhancing the livestock productivity [2,3].

With the continuous development of animal husbandry, many local sheep breeds in China have exhibited excellent and unique genetic traits. For example, the small-tail Han sheep and Hu sheep, known for their high fertility, have become the most ideal maternal parents in the current domestic breeding process of meat sheep and the main breeds for intensive housing feeding [4,5]; Lanzhou large-tailed sheep, which have a coarse hair type and are a meat and fat dual-purpose breed, are tall in the early stages, but also have the characteristics of strong adaptability, good meat quality, and roughage tolerance. However, the number of Lanzhou big-tail sheep is very small and the breed is nearing extinction [6]. TS is an excellent local fat-tail breed with semi-fine hair. It has the advantages of thin skin and fine bone, good meat production, excellent wool quality, and rapid growth and development [7].

The efficiency of lamb production primarily depends on the growth rate of sheep, which is a complex trait consisting of BW, body length (BoL), daily weight gain, and other related characteristics. These traits can be influenced by various factors, including genetics, nutrient levels, environmental conditions, and others [8]. The influence of genetics on growth performance has been well-documented in the literature [9,10,11]. The cost of genetic marker detection has significantly decreased in recent years, making it possible to analyze the presence of different markers in individuals simultaneously [12]. DNA markers, such as single nucleotide polymorphisms (SNPs), insertions/deletions (InDels), and copy number variants (CNVs), have been widely utilized in MAS (Marker-Assisted Selection) [13,14,15].

InDel is a phenomenon characterized by the presence of insertions or deletions resulting from homologous alignments [16]. In the development of InDel markers for functional genes, several InDels have been identified as being associated with traits related to the growth of mutton sheep [17]. Regulatory genes typically function within intricate networks of upstream and downstream signaling pathways. InDel variation may affect the normal functioning of an entire signaling pathway by altering gene expression or modifying the structure and function of proteins. Therefore, studying InDel variation in signaling pathways helps to uncover the interactions and regulatory relationships between these genes, thus revealing the genetic mechanisms underlying production traits.

The Hippo pathway is an evolutionarily conserved signaling cascade with extensive upstream regulatory effects. It plays a crucial role in cellular misperception, physical signaling within the plasma membrane, and regulating responses through the control of cell differentiation and proliferation [18]. One study has confirmed that the Hippo signaling pathway can influence reproduction by regulating early embryonic development in the ovine oviduct [19]. The activity of the Hippo pathway strongly depends on various factors, such as cellular connectivity, cellular structure, and the mechanical properties of the microenvironment [20]. Furthermore, numerous upstream factors impact the activation of core Hippo pathway kinases and YAP (Yes-associated protein). These upstream biochemical signals and crosstalk from other major signaling pathways include Ds-Fat signaling, epidermal growth factor receptor (EGFR), Wnt signaling, bone morphogenetic proteins (BMP), Hedgehog, and G-protein-coupled receptors (GPCRs), as well as integrin signaling, cell polarization, and cell metabolic pathways [21]. The Hippo signaling pathway plays an important role in energy metabolism [22]. In mammals, the Hippo kinase cascade consists of a STE20-like kinase (MST1/2) and a large tumor suppressor kinase (LATS1/2). The outcomes of LATS-mediated phosphorylation pathways include YES1-associated transcriptional regulatory factor (YAP1) and WW-domain-containing transcriptional regulatory factor 1 (TAZ), which are involved in various biological processes [18,23]. Moreover, the Hippo pathway has been shown to play an important role in controlling organ size in mammals [24]. Data indicate that the Hippo pathway is involved in regulating ovaria follicle growth and development. Studies have demonstrated that the expression levels of *MST1/2* and *LATS1/2* in this pathway decrease, while the expression of *YAP1* increases as follicle diameter increases [23]. This suggests that these genes are closely related to reproduction. However, there are few studies on the effects of polymorphisms and InDels of the Hippo signaling pathway on growth traits in sheep.

To address this question, this study focused on sheep (TS, HS, LLTS, STHS) as the research subjects and used public databases to screen for potential InDels in the Hippo signaling pathway, specifically in the *MST1/2*, *LATS1/2*, *SAV1*, *MOB1A/B*, and *YAP/TAZ* genes. InDel technology was employed to identify existing InDels in sheep, and the relationship between the polymorphisms of key genes in this pathway and growth traits was analyzed. Additionally, key InDel molecular markers affecting growth and development were identified for use in auxiliary selection. The purpose of this study was to fill the gap in understanding the contribution of key genes in the Hippo pathway to sheep reproduction and growth. Ultimately, this research aimed to assist breeders in more accurately evaluating the potential of production traits in sheep, thereby accelerating the breeding process of local sheep breeds in China and improving breeding efficiency.

## 2. Materials and Methods

### 2.1. Ethics Statement

The use of animals and all experimental protocols were approved by the institution Animal Care and Use Committee (lACUC) of the Northwest A&F University (Yangling, Shaanxi, China).

### 2.2. Animal Samples and Data Collection

To explore the polymorphisms of key genes in the Hippo signaling pathway in sheep, 618 samples from four local sheep breeds in China were selected: Lanzhou large-tailed sheep (LLTS, *n* = 61; Yongjing, Gansu Province, China), small-tail Han sheep (STHS, *n* = 190; Yongjing, Gansu Province, China), Hu sheep (HS, *n* = 201; Mengjin, Henan Province, China), and Tong sheep (*n* = 166; Baishui, Shanxi Province, China). These samples were all from commercial farms. The samples were sheep of the same breed at the same developmental stage. All animals were adults, healthy, and unrelated. All tissue samples collected were quickly frozen in liquid nitrogen and stored at −80 °C for preservation. Genomic DNA was extracted from blood or ear tissue (610 using blood, 8 using ear tissue). Growth traits, including height at hip cross (HiC), rump length (RuL), rump height (RuH), rump width (RuW), body length (BoL), body oblique length (BOL), body height (BoH), hip width (HiW), chest width (ChW), chest depth (ChD), chest circumference (ChC), cannon circumference (CaC), and body weight (BW), were recorded.

### 2.3. Genomic DNA Isolation and DNA Pool Construction

Genomic DNA samples were isolated from whole blood using the DN02-Small Volume Whole Blood Genomic DNA Extraction Kit (Aidlab, Beijing, China), and the DNA quality and purity of each sample were assessed using a Nanodrop 1000 (Thermo Scientific, Waltham, MA, USA). Each sample was then diluted with ddH_2_O to a standard concentration of 50 ng/µL and stored at −20 °C for subsequent genotyping. Thirty DNA samples were randomly selected from each breed to construct the genomic DNA library. The genomic DNA bank was used as a PCR template to detect possible InDel sites in the Hippo signaling pathway of sheep.

### 2.4. Primer Design, PCR Amplification, and Indel Genotyping

Nine genes (*MST1*, *MST2*, *LATS1, LATS2*, *YAP, TAZ*, *SAV1*, *MOB1A*, and *MOB1B*) of the Hippo signaling pathway that had not yet been validated in sheep were selected. Based on data from the Ensembl database (http://lasia.ensembl.org/index.html, format: 12 February 2021), primers for InDels with fragment sizes ≥ 5 bp or located in essential positions were designed using NCBI Primer-BLAST (https://www.ncbi.nlm.nih.gov/tools/primer-blast/) or Primer Premier software (Version 5.0, PREMIER Biosoft International, Palo Alto, Santa Clara, CA, USA) (Table 1).

The touchdown polymerase chain reaction (TD-PCR) technique was used to assess the polymorphisms during the genotyping process. The procedure was as follows: TD-PCR was performed in a total volume of 15 μL, consisting of 0.5 μL genomic DNA, 0.5 μL forward and reverse primers, 7.5 μL 2X SanTaq PCR Master Mix (with Blue Dye) (Sangon Biotech, Shanghai, China), and 7.5 μL ddH2O. The amplification conditions included initial denaturation at 95 °C for 3 min, followed by denaturation at 95 °C for 15 s, annealing at 60 °C for 15 s (with a 1 °C reduction per cycle over 20 cycles), and extension at 72 °C for 30 s. This was followed by denaturation at 95 °C for 30 s, annealing at 53 °C for 30 s, and extension at 72 °C for 30 s, for a total of 25 cycles. Finally, the reaction was extended for 10 min at 72 °C and stored at 4 °C. The products were analyzed by 3.5% agarose gel electrophoresis at a constant voltage of 120 V for 1 h. PCR products that were correctly identified were sent for sequencing (Sangon Biotech Co., Ltd., Xi’an, China).

### 2.5. Estimation of Population Genetic Parameters and Statistical Analysis

The method provided by Botstein et al. (1980) was used to calculate the genotype and allele frequencies of InDel mutations in four sheep breeds. Haploview software was used to analyze linkage disequilibrium. The online Genetic Diversity Index calculator and GenAlEx 6.5 were used to calculate homozygosity (Ho), heterozygosity (He), effective allele numbers (Ne), polymorphism information content (PIC), and Hardy–Weinberg equilibrium (HWE). Ho and He are indicators of genetic variation in a population, while PIC is an indicator of population polymorphism.

We used the linear statistical model: Y_ijk_ = µ + A_i_ + B_j_ + G_k_ + E_ijk_, where Y_ijk_ represents the observation of growth-related traits; µ represents the population average for each trait; A_i_ represents the effect of age; B_j_ represents the influence of sex; G_i_ represents the fixed effect of genotype; E_ijk_ stands for random residual [12]. The fixed effect of genotype was the main factor contributing to the difference in growth traits. A *p*-value of less than 0.05 was considered significant. Independent sample t-tests and one-way analysis of variance (ANOVA) were performed using SPSS 24 software to determine the association between InDel sites and growth traits.

## 3. Results

Screening the key genes of the Hippo signaling pathway in the database, a total of 154 potential InDel loci were screened, and after the PCR amplification and sequencing, 23 InDel loci were identified in the four sheep breeds. Among them, there were three *MST1* genes, seven *MST2* genes, eight *YAP* genes, two *TAZ* genes, and three *MOB1A* genes, while no InDel sites were found in *MOB1B*, *LATS1/2*, and *SAV1*. In addition, 12 InDel loci were associated with sheep production traits.

### 3.1. Identification and Analysis of InDel Loci of MST1/2 Gene

#### 3.1.1. Identification of InDel Variants in the *MST1/2* Gene

After PCR amplification, agarose gel electrophoresis isolation and sanger sequencing verification, three InDel sites of the *MST1* gene were identified in four sheep breeds, among which MST1-S2 and MST1-S17 InDel sites were found in four sheep breeds, and MST1-S8 was found in TS and HS. A total of seven InDel loci were identified in MST2 gene, among which four InDel loci, MST2-S1, MST2-S4, MST2-S27, and MST2-S38, were all present in four sheep breeds. Two InDel sites, MST2-S36 and MST2-S40, exist in STHS, TS, and HS, while MST2-S15 exists only in LLTS (Supplementary Figure S1).

**Figure 1:**
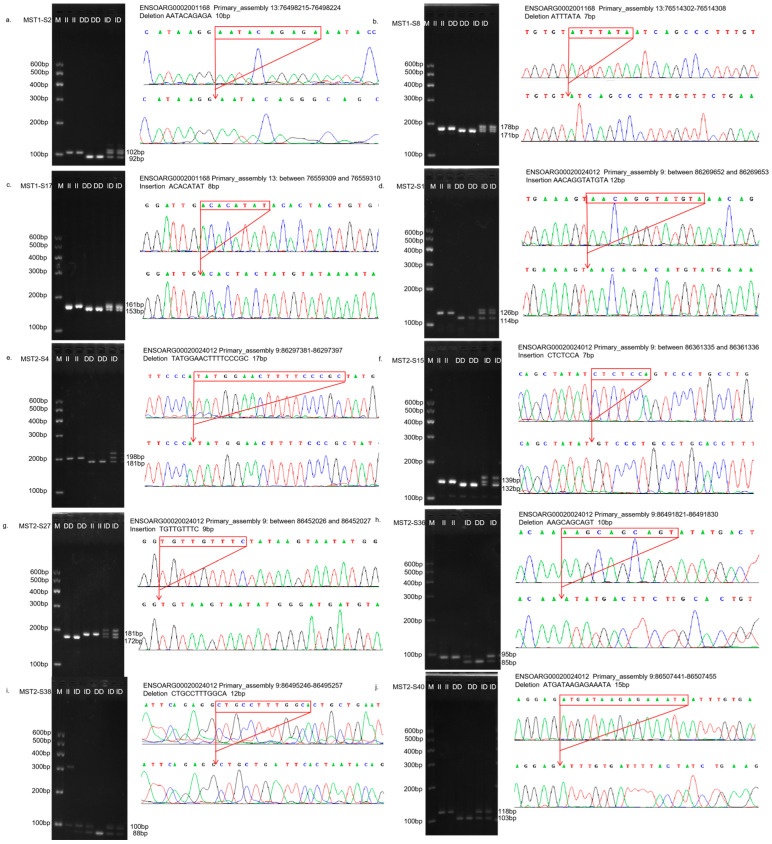
The electrophoresis pattern and sequence chromatograms of the *MST1/2*. (**a**–**c**) Agarose gel electrophoresis and sequencing peaks of MST1-S2, MST1-S8, and MST1-S17 InDel loci detected in sheep *MST1* gene; (**d**–**j**) Agarose gel electrophoresis and sequencing peaks of MST2-S1, MST2-S4, MST2-S15, MST2-S27, MST2-S36, MST2-S38, and MST2-S40 InDel loci detected in sheep *MST2* gene. M represents Marker, II represents insertion homozygote, ID insertion deletion heterozygote, DD deletion homozygote.

The InDel loci of the identified *MST1* and *MST2* genes were typed. The relevant information of agarose gel electrophoresis typing and sequencing of all InDel loci is shown in Figure 1. The MST1-S2 (rs1087487367), MST1-S8 (rs594737589), and MST1-S17 (rs592335858) loci were all consistent with those predicted in the Ensembl database. MST2-S1 (rs590524333), MST2-S4 (rs1089390863), MST2-S15 (rs590246822), MST2-S27 (rs602979791), MST2-S36 (rs594841887), and MST2-S38 (rs605961263) were all consistent with those predicted in the Ensembl database. It should be noted that the actual deletion in MST2-S40 (rs605179432) comprises the sequence ATGATAAGAGAAATA, totaling 15 bp. However, the deletion sequence recorded in the database was ATGTTCCTGG, which was 10 bp long.

#### 3.1.2. Genetic Parameter Analysis of InDel Loci of *MST1/2* Gene in Sheep

Indel *MST1/2* gene loci genotypes, allele frequencies, and associated polymorphism information are shown in Table 2 and Table 3. In MST1-S2, the dominant genotype was type II for LLTS, STHS, and HS, while it was DD for TS. At the MST1-S8 locus, the II genotype was dominant in both TS and HS. For the MST1-S17 locus, the DD genotype was the most common across all four breeds.

Among the InDel loci of the *MST2* gene, the dominant genotype of MST2-S1 was DD. For MST2-S4 and MST2-S15, the dominant genotype in the HS breed was DD, while the other three breeds predominantly exhibited the ID genotype. The dominant genotypes of MST2-S36, MST2-S38, and MST2-S40 were all Type II. In contrast, MST2-S37 had a dominant LLTS-type ID genotype, whereas the other three breeds were predominantly Type II.

The InDel sites of *MST1* ranged from 0.187 to 0.422 for Ho and from 0.178 to 0.441 for He. When the Ne range was from 1.217 to 1.788, the distribution of alleles at MST1-S20 locus was the most uneven in TS (Ne = 1.217), and that at MST1-S2 locus was the most uniform in HS (Ne = 1.788). Except for the MST1-S10 site of TH, which exhibited low polymorphism (PIC < 0.25), all other sites displayed moderate polymorphism (0.25 < PIC < 0.5).

#### 3.1.3. Linkage Disequilibrium Analysis for InDel Loci in the *MST1/2* Gene

**Figure 2:**
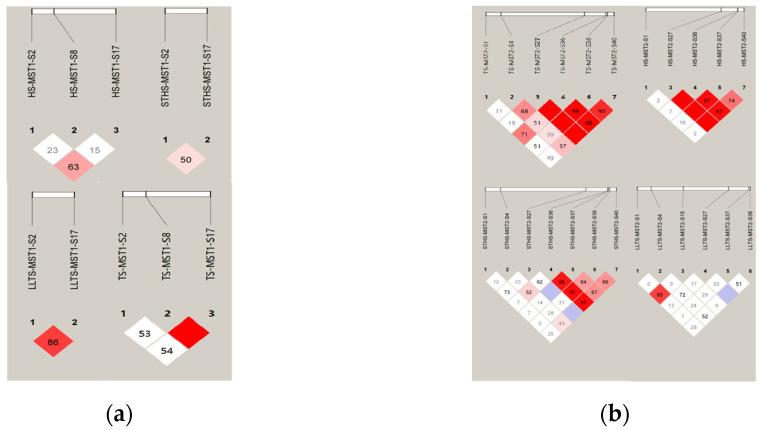
LD analysis of five InDels of MST1/2 in four populations. (**a**) D’ analysis of MST1 InDels in four breeds of sheep; (**b**) D’ analysis of MST2 InDels in four breeds of sheep. Note: The darker the color of the table, the higher the degree of linkage.

The linkage disequilibrium (LD) analysis of the *MST1* and *MST2* genes was performed using Haploview 4.2, as shown in Figure 2. The results show that MST1-S2 and MST1-S17 and MST2-S1 and MST2-S4 were strongly linked in LLTS. In STHS, MST2-S36 loci were strongly linked to MST2-S37, MST2-S38, and MST2-S40, respectively. In TS, MST1-S8 sites were strongly linked to MST1-S20 and MST2-S27 sites were strongly linked to MST2-S38 and MST2-S40, and MST2-S36 sites were strongly linked to MST2-S38 and MST2-S40, respectively. MST2-S38 and MST2-S40 were in a strong linkage state. In TS, MST2-S27 sites were strongly linked to MST2-S36 and MST2-S40, and MST2-S36 sites were strongly linked to MST2-S40.

#### 3.1.4. Association Between InDel Loci and Growth Traits in Sheep *MST1/2* Gene

Analysis of the associations between single InDel mutation sites and growth traits revealed that, in the *MST1* gene, individuals with the II genotype at the MST1-S2 locus of HS had a significantly greater influence on BoH compared to those with the ID genotype (*p* < 0.05), while the difference was not significant for the DD genotype (*p* > 0.05). The influence of the II genotype on BOL was significantly higher than that of the DD genotype (*p* < 0.05). DD type mutations at MST1-S8 significantly decreased the maximum forehead width (MFoW) of TS (*p* < 0.05). Type II mutations at MST1-S17 significantly reduced BoL and head depth (HeD) of TS (*p* < 0.05) and CaC and BW of HS (*p* < 0.05), as shown in Table 4.

In the *MST2* gene, mutations at the MST2-S1 site significantly increased the BW of HS (*p* < 0.05). Type II mutation at MST2-S4 significantly increased BOL, BoH, and Hih of LLTS (*p* < 0.05), but significantly decreased the CaC of STHS (*p* < 0.05). The type II mutation of MST2-S15 significantly decreased the BW and ChC of LLTS (*p* < 0.05). The DD mutation at MST2-S36 significantly decreased the BoH of STHS, but significantly increased the CaC of TS (*p* < 0.05). DD type mutations at MST2-S37 significantly reduced the BoH and Hih of STHS (*p* < 0.05), as shown in Table 5.

### 3.2. Identification and Analysis of InDel Loci of YAP/TAZ Gene

#### 3.2.1. Identification of InDel Variants in the *YAP/TAZ* Gene

A total of eight InDel sites in the *YAP* gene were identified across four sheep breeds. These included the YAP-S4, YAP-S5, and YAP-S7 loci in LLTS, STHS, and HS; the YAP-S11 locus in LLTS and HS; and the YAP-S15 locus in LLTS and STHS. Additionally, three InDel sites—YAP-S17, YAP-S19, and YAP-S21—were found in all four sheep breeds. In contrast, InDel sites in the *TAZ* gene were only identified in LLTS and TS sheep, specifically the TAZ-S17 and TAZ-S24 loci. The TAZ-S17 locus was present in both LLTS and TS breeds, while the TAZ-S24 locus was found exclusively in the TS breed (Supplementary Figure S2).

**Figure 3:**
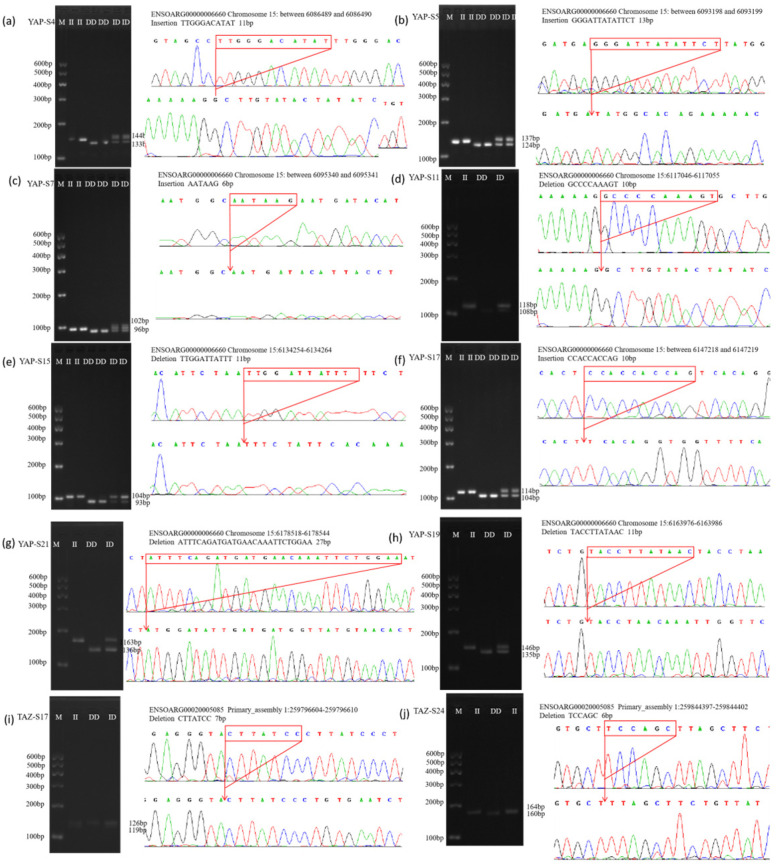
The electrophoresis pattern and sequence chromatograms of the *YAP* and *YAZ*. (**a**–**h**) Agarose gel electrophoresis and sequencing peaks of InDel loci detected in *YAP* gene in sheep; (**i**,**j**) Agarose gel electrophoresis and sequencing peaks of InDel loci detected in *TAZ* gene in sheep. M represents Marker, II represents insertion homozygote, ID insertion deletion heterozygote, DD deletion homozygote.

The results of agarose gel electrophoresis and sequencing for all InDel sites in the *YAP/TAZ* genes detected across the four sheep breeds are presented in Figure 3. These loci were consistent with those predicted in the Ensembl database.

#### 3.2.2. Genetic Parameter Analysis of InDel Loci of *YAP/TAZ* Gene in Sheep

Table 6 shows the genotype and allele frequency and polymorphism information of InDel sites of *YAP* and *TAZ* genes. In the *YAP* gene, the dominant genotypes of YAP-S4, YAP-S5, and YAP-S7 locus in LLTS, STHS, and HS were the DD genotype, DD genotype, and II genotype, respectively. The II genotype was dominant at YAP-S11 in LLTS and HS, at YAP-S15 in LLTS and STHS, and at YAP-S17 in LLTS and TS, while the ID genotype was dominant at YAP-S17 in STHS and HS. Type II at YAP-S19 and YAP-S21 was the dominant genotype of all four sheep breeds. For the *TAZ* gene, the ID genotype was dominant at TAZ-S17 in LLTS and TS, and the II genotype was dominant at TAZ-S24 in TS.

The InDel sites of *YAP* ranged from 0.045 to 0.516 for observed heterozygosity (Ho) and from 0.043 to 0.494 for expected heterozygosity (He). The number of effective alleles (Ne) ranged from 1.045 to 1.978, with the HS YAP-S11 locus exhibiting the greatest allele unevenness (Ne = 1.045) and the STHS YAP-S17 locus showing the most uniform distribution (Ne = 1.978). Among these InDel sites, those with low polymorphism (PIC < 0.25) included the YAP-S4, YAP-S7, YAP-S11, and YAP-S19 sites in LLTS; the YAP-S15 and YAP-S19 sites in STHS; and the YAP-S17, YAP-S19, and YAP-S21 sites in TS, as well as the YAP-S11, YAP-S19, and YAP-S21 sites in HS. Sites that were moderately polymorphic (0.25 < PIC < 0.5) are indicated in Table 6. The InDel sites in the *TAZ* gene ranged from 0.331 to 0.443 for Ho and from 0.315 to 0.500 for He, with Ne ranging from 1.460 to 1.998. LLTS and TS displayed the most evenly distributed alleles at the TAZ-S17 locus (Ne = 1.998). These two loci, YAP-S17 and TAZ-S17, might be less influenced by genetic drift. As shown in Table 6, two loci (TAZ-S17 and TAZ-S24) exhibited moderate polymorphism (0.25 < PIC < 0.5).

#### 3.2.3. Linkage Disequilibrium Analysis for InDel Loci in the *YAP* and *TAZ* Genes

**Figure 4:**
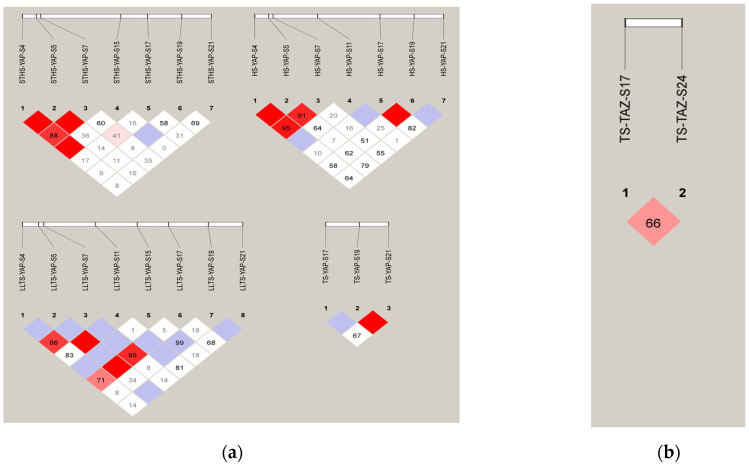
The electrophoresis pattern and sequence chromatograms of *YAP* and *YAZ*. (**a**) D’ analysis of *YAP* InDels in four breeds of sheep; (**b**) D’ analysis *TAZ* InDels in four breeds of sheep. Note: The darker the color of the table, the higher the degree of linkage.

In LLTS, YAP-S5 exhibited strong linkage with both YAP-S11 and YAP-S17. In STHS, the YAP-S4 site was strongly linked to the YAP-S5 and YAP-S15 sites, while YAP-S5 and YAP-S7 sites showed strong linkage with each other. Other sites displayed weak linkage. In TS, a strong linkage was observed between the YAP-S19 and YAP-S21 sites. In HS, YAP-S4 showed strong linkage with both YAP-S5 and YAP-S7, and the YAP-S5 and YAP-S7 sites, as well as the YAP-S17 and YAP-S19 sites, were strongly linked. Other sites showed weak linkage. The TAZ-S17 and TAZ-S24 sites in TS were weakly linked, as shown in Figure 4.

#### 3.2.4. Association Between InDel Loci and Growth Traits in Sheep *YAP/TAZ* Gene

Association analysis was conducted between the InDel sites of *YAP* and *TAZ* genes and the growth traits of the four breeds of sheep (Table 7). For the YAP-S19 locus, the ID genotype in LLTS had significantly higher effects on ChD and BoH compared to the II genotype (*p* < 0.05). In TS, the DD genotype at YAP-S19 significantly enhanced BoL more than other genotypes (*p* < 0.05). The II genotype at YAP-S21 in LLTS and STHS showed a significantly higher effect on ChC compared to the DD genotype (*p* < 0.05). In TS, the ID genotype at YAP-S21 exhibited superior RuH, HeL, and CaC compared to the II genotype (*p* < 0.05). Unfortunately, mutations in YAP-S4, YAP-S5, YAP-S7, YAP-S11, YAP-S15, YAP-S17, TAZ-S17, and TAZ-S18 loci were not significantly associated with growth traits in sheep (*p* < 0.05).

### 3.3. Identification and Analysis of InDel Loci of the MOB1A/B Gene

#### 3.3.1. Identification of InDel Variants in the *MOB1A/B* Gene

After verification, we screened out InDel mutations of the *MOB1A/B* gene in four sheep breeds. As shown in Supplementary Figure S3 among the three InDel sites of the *MOB1A* gene, MOB1A-S4 sites exist in LLTS and STHS, and MOB1A-S7 sites exist in all four sheep breeds. MOB1A-S10 sites exist in LLTS, STHS, and HS. Unfortunately, none of the nine potential InDel sites of the MOB1B gene screened in the database were identified in any of the four sheep breeds.

**Figure 5:**
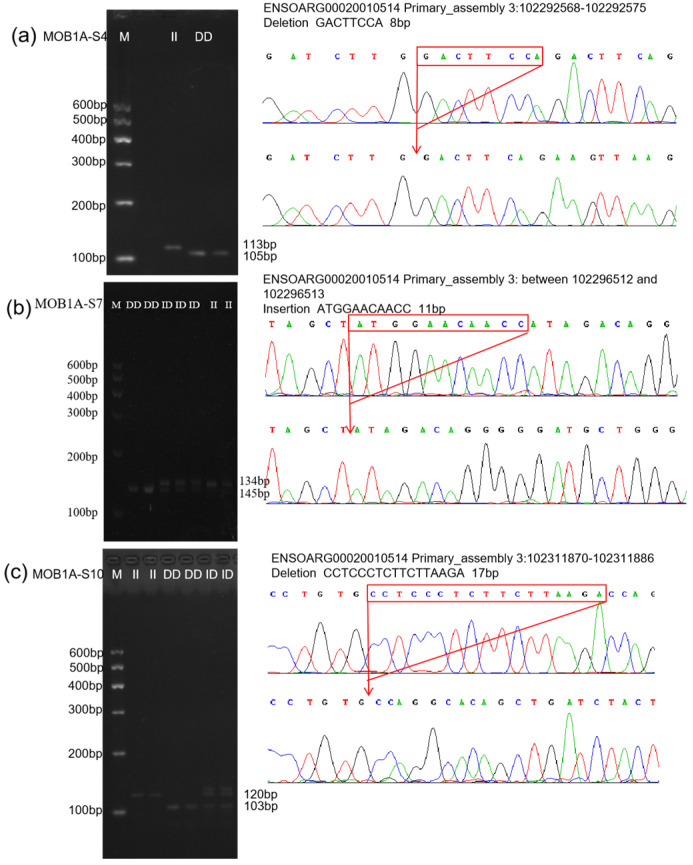
The electrophoresis pattern and sequence chromatograms of the MOB1A. (**a**–**c**) Agarose gel electrophoresis and sequencing peaks of MOB1A-S4, MOB1A-S7, and MOB1A-S10 InDel loci detected in sheep MOB1A gene. M represents Marker, II represents insertion homozygote, ID represents insertion deletion heterozygote, DD represents deletion homozygote.

All individuals of the four sheep breeds were typed according to the InDel sites of the identified *MOB1A* gene. The agarose gel electrophoresis typing images and sequencing results for all InDel sites are shown in Figure 5. The genotyping results for the MOB1A-S4 (rs605284550), MOB1A-S7 (rs595273936), and MOB1A-S10 (rs1094858631) loci were consistent with those predicted in Ensembl.

#### 3.3.2. Genetic Parameter Analysis of InDel Loci of the *MOB1A* Gene

The frequency and polymorphism information for the genotypes and alleles at the InDel sites of the *MOB1A* gene in the four sheep breeds are presented in Table 8. At the MOB1A-S4, MOB1A-S7, and MOB1A-S10 loci, genotype II was the dominant genotype, and allele I was the dominant allele.

The Ho of InDel sites of the *MOB1A* gene ranged from 0.137 to 0.433, and He ranged from 0.172 to 0.453. The Ne range was from 1.208 to 1.827, in which STHS had the most uneven allele distribution at the MOB1A-S4 locus (Ne = 1.208), and HS had the most uniform allele distribution at the MOB1A-S7 locus (Ne = 1.827). All three InDel sites in sheep showed low polymorphism (PIC < 0.25) except the MOB1A-S7, which was moderately polymorphic (0.25 < PIC < 0.5) (Table 8).

#### 3.3.3. Genetic Parameter Analysis of InDel Loci of *MOB1A* Gene

**Figure 6:**
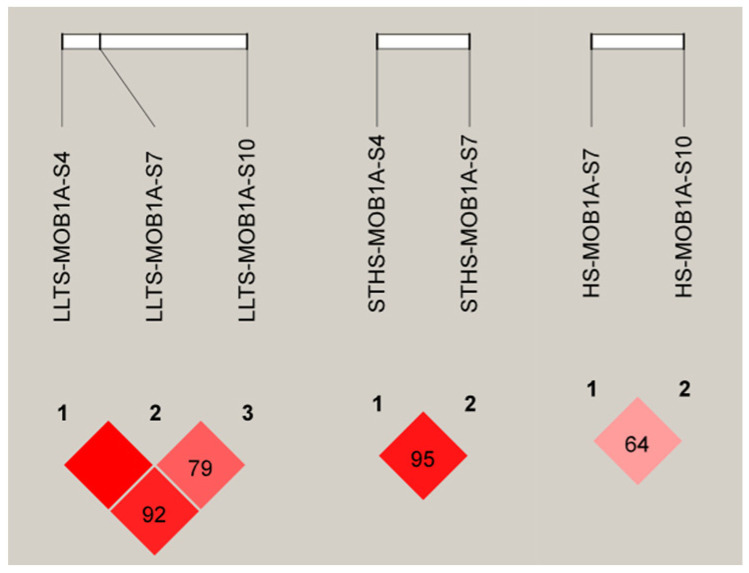
D’ analysis of *MOB1A* gene InDel locus. Note: The darker the color of the table, the higher the degree of linkage.

In LLTS, MOB1A-S4 exhibited strong linkage with both MOB1A-S7 and MOB1A-S10. In STHS, strong linkage was observed between the MOB1A-S4 and MOB1A-S7 sites. However, both InDel sites of the *MOB1A* gene in HS were weakly linked, as shown in Figure 6.

#### 3.3.4. Association Between InDel Loci in the *MOB1A* Gene and Growth Traits in Sheep

The InDel site of the *MOB1A* gene was associated with the growth traits of four sheep breeds. The mean CaC of sheep with the DD genotype at the MOB1A-S4 locus of STHS was significantly higher than that of the ID genotype and II genotype (*p* < 0.05); the mean CaC of the DD genotype at MOB1A-S7 was significantly higher than that of the II genotype (*p* < 0.01), and the BW of the DD genotype was significantly higher than that of the II genotype (*p* < 0.05). The HiW and HeL of DD genotype at TS MOB1A-S7 were significantly higher than those of the ID genotype (*p* < 0.05). The CaC of DD and ID genotype at MOB1A-S10 in HS was significantly higher than that of the II genotype (*p* < 0.05), but the CaC of the II genotype at MOB1A-S10 in LLTS was significantly higher than that of the ID genotype (*p* < 0.05). As shown in Table 9, the ChC of the DD and ID genotypes at MOB1A-S10 in STHS was significantly higher than that of the II genotypes (*p* < 0.05).

## 4. Discussion

The Hippo signaling pathway regulates a variety of biological processes, including cell growth and fate determination, organ development and regeneration [25]. Research showed that there are many genes related to the Hippo signaling pathway in the long muscle tissue of the back and meridian ridge of the Liaoning Cashmere goat [26]; in addition, studies involving the ovaries of dairy cattle have shown that the YAP-mediated transcriptional activity of preovulatory granulosa cells plays a key role in LH-induced ovulation in single-ovulating species [27]. Previous studies have mainly focused on the influence of key genes in the Hippo pathway on cell proliferation, immunity, and inflammation [28,29,30]. However, few studies have reported systematically on the relationship between InDel polymorphisms in key genes of the Hippo signaling pathway and growth traits in sheep. This study analyzed the relationship between Hippo signaling key gene polymorphisms and growth traits in 618 sheep. Thirteen InDels related to production traits were screened in *MST1/2*, *LATS1/2*, *YAP/TAZ*, *MOB1A/B*, and *SAV1* genes of HS, STHS, LLTS, and TS. InDel variation in this signaling pathway helps to reveal the interaction and regulatory relationship between these genes. To provide basic data for breeding of LLTS, STHS, TS, and HS, we conducted comprehensive genetic analyses.

*MST1/2* was originally reported as a key kinase in the Hippo pathway, which regulates cell proliferation, apoptosis, and differentiation by inhibiting *YAP* nuclear translocation [31]. Moreover, as transcription co-activators of the Hippo pathway, the activity of *YAP/TAZ* sustains the self-renewal and tumor-initiation capabilities of cancer stem cells [32]. Li et al. [33] studied SNP located near genes significantly related to digestive traits in Suhuai pigs and found that *MST1* and *LATS1* could be used as candidate genes related to intestinal homeostasis and health function. The data showed that genes such as *MST1* may play an important role in regulating the immunity and egg production rate of hens after maternal lipopolysaccharide (LPS) stimulation [34]. Similarly, Cai et al. [35] used the combined analysis of WGS and RNA-seq data to screen and identify important potential candidate genes for egg production of *MAST2* chickens. In our study, the association analysis between the 12 *MST1/2* InDel sites screened in this study and production traits revealed that MST1-S1/S4/S2/S8/S17 and MST2-S15/S36/S37 were significantly associated with the growth traits of sheep (such as BoH, ChC, CaC, BW, BOL), suggesting that *MST1/2* could be used as an important candidate gene for research on production traits of livestock and poultry. Liu et al. [36] reported that, at the 5 bp and 9 bp InDel sites of the IGF2BP1 gene in sheep, individuals with heterozygous (ID) genotypes exhibited superior growth performance, particularly for BW. Zhang et al. [37] investigated the genetic variation in Chaka sheep and STHS, focusing on the LRRC8B genes. Their study identified both ID and DD genotypes associated with an insertion/deletion (InDel) polymorphism in the LRRC8B gene. Notably, they found a significant association between this InDel in the LRRC8B gene and the breast depth of STHS (*p* < 0.05). Sheep with the ID genotype showed better production traits than those with the DD genotype, and Wu found that, in the FGF7 gene, goats with the ID and/or II genotypes have superior growth traits compared to those with the DD genotype [38]. Additionally, our research demonstrated that the insertions or deletions (InDel) can contribute to differences in livestock production traits. It is worth noting that our study’s findings were contrary to Wu’s research results. In our study, the DD genotype exhibited superior growth traits. For example, at the MOB1A-S4 site, STHS sheep with the DD genotype had a significantly greater cannon circumference (CaC) than sheep with the other genotypes. We speculated that different genes played distinct roles in animal growth. The two InDel sites of MOB1A-S7 and MOB1A-S10 showed similar results. Additionally, we speculated that the reason for the difference in the dominant genotypes between our experiment and those reported by Wu et al. might have been due to differences in sheep breeds.

Genetic diversity analysis is also an important part of understanding variation. By analyzing the levels, spatial and temporal distributions of genetic variation within species, and its relationship with environmental conditions, we can better understand and more effectively protect valuable animal resources [39]. The genotypic distribution of all InDel loci detected by us were in line with the Hardy–Weinberg law, in which MST1-S2/S4, MST2-S15, and TAZ-S17 had higher effective number of alleles than other loci in the four sheep breeds, and were moderately polymorphic (0.25 < PIC < 0.50). It may be because these four InDel loci not only exhibit uniform allele distribution within the population but also show substantial variation, leading to stronger selection pressures. Therefore, the genetic diversity at the InDel sites of key Hippo signaling pathway genes that we have identified can serve as valuable genetic markers for breeding sheep with ideal growth traits.

## 5. Conclusions

We identified 23 InDel sites in key genes of the Hippo signaling pathway across four breeds of sheep. There were three InDel sites in the *MST1* gene, seven InDel sites in the *MST2* gene, eight InDel sites in the *YAP* gene, two InDel sites in the *TAZ* gene, and three InDel sites in the *MOB1A* gene. All 25 InDel loci were in the Hardy–Weinberg equilibrium state, among which the MST1-S2/4, MST2-S15, and TAZ-S17 loci exhibited the highest genetic diversity. Notably, three of the *MST1*, four of the *MST2*, two of the *YAP*, and three of the *MOB1A* InDel loci were significantly associated with the growth traits of sheep. Our findings imply that these 12 InDel loci can be utilized as valuable DNA markers to enhance the growth traits of local Chinese breeds of sheep.

## Figures and Tables

**Table 1:** T InDel locus primers of key genes in the Hippo signaling pathway.

Gene	Locus	Primer Sequence	Product Size	InDel Size
*MST1*	MST1-S2	F:CATGCCTCTAAAGATGAG	102 bp	10 bp
		R:CAGACTCCGGGAGGACAA		
	MST1-S8	F:GGGCTTTGGTTCTCAGGC	178 bp	7 bp
		R:GTGGGACTACTTCACCATCTGT		
	MST1-S17	F:AAGAGCAAGCCTTCAGCG	153 bp	8 bp
		R:CAGCAGGTTCTCACTCGTTA		
*MST2*	MST2-S1	F:CTACATGCAATCTCAAGT	114 bp	12 bp
		R:TAAGAAGTTCAAGGTAAT		
	MST2-S4	F:TTTTCAGTAGTAAGGCTGTT	181 bp	17 bp
		R:CGACCCGTAGTAGGATTT		
	MST2-S15	F:GCCACTTCCAGCTTTGGG	132 bp	7 bp
		R:ACAGACAGAAGAGCGAAA		
	MST2-S27	F:TATGAACTGCCTGGTGAA	172 bp	9 bp
		R:AGGGTGGAAACTATAACC		
	MST2-S36	F:TTCTTCCGAAGGGCACAA	95 bp	10 bp
		R:TCATTTTACACCATCAGC		
	MST2-S38	F:GTTATGGAACTACTACTATTAC	100 bp	12 bp
		R:GGGGTTTGATCTCAATCT		
	MST2-S40	F:AGTTGGTTGTTTCCTGTT	118 bp	10 bp
		R:TTCAGTTAGCCTCAATAC		
*YAP*	YAP-S4	F:CTGGCATAGCTGTTTTAA	133 bp	11 bp
		R:TAGACATTTGTTCGGGAT		
	YAP-S5	F:AACTTTAGCTTTTGTCACTC	124 bp	13 bp
		R:AAGGGCAGATTCTAACATTA		
	YAP-S7	F:GATCAAACTATGGATTGT	96 bp	6 bp
		R:AGATGTCTGAAAAGGTAA		
	YAP-S11	F:GAAACCCAACTGAAGAAAGG	118 bp	10 bp
		R:ATTTGCACAAAGAAATACCC		
	YAP-S15	F:AAGCTATTGTCATCTACAAC	104 bp	11 bp
		R:CAGTAAACAATATCTAGGGA		
	YAP-S17	F:TACAAAGCAACTAACAACG	104 bp	10 bp
		R:TAGTGCAACTGCTGAAAA		
	YAP-S19	F:CTCCCTTTTCATAATCCACT	146 bp	11 bp
		R:CAGAATCTTGGCAAAGTTGA		
	YAP-S21	F:AGACTAAAGCCCCACCTCCC	163 bp	27 bp
		R:GTGGCATCTAAGTTCATTCA		
*TAZ*	TAZ-S17	F:GACACTACAGCTCCAGCAT	126 bp	7 bp
		R:TCAGGGACCAGATTCACA		
	TAZ-S24	F:TGGAGGTAGGAGGTGAGA	164 bp	6 bp
		R:CAAGGCTGGTCTAGGATG		
*MOB1A*	MOB1A-S4	F:CTGTAAACCAGGAATAGG	113 bp	8 bp
		R:CTGGAGGTCTCATAAGCA		
	MOB1A-S7	F:ACAATGGCAACTGAGCTTTC	145 bp	11 bp
		R:ATGGGGTATCTTTTGGGAGG		
	MOB1A-S10	F:TTTTCCCCAATACGTCCC	120 bp	17 bp
		R:CAGATTAGGCAAAGTCAT		

**Table 2:** Polymorphism information of InDel locus of sheep *MST1* gene.

Locus	Breed	Number	Genotypic Frequency			Allelic Frequency		Polymorphism Information				
		n	II	ID	DD	I	D	Ho	He	Ne	PIC	$x^{2}$
MST1-S2	LLTS	61	0.541	0.344	0.115	0.713	0.287	0.344	0.409	1.693	0.326	*p* > 0.05
	STHS	190	0.495	0.416	0.089	0.703	0.297	0.416	0.418	1.717	0.330	*p* > 0.05
	TS	166	0.060	0.422	0.518	0.271	0.729	0.422	0.395	1.653	0.317	*p* > 0.05
	HS	201	0.478	0.388	0.134	0.672	0.328	0.388	0.441	1.788	0.344	*p* > 0.05
MST1-S8	TS	166	0.530	0.386	0.084	0.723	0.277	0.386	0.401	1.668	0.320	*p* > 0.05
	HS	201	0.672	0.274	0.055	0.808	0.192	0.274	0.310	1.450	0.262	*p* > 0.05
MST1-S17	LLTS	61	0.016	0.344	0.639	0.189	0.811	0.344	0.307	1.442	0.260	*p* > 0.05
	STHS	190	0.042	0.295	0.663	0.189	0.811	0.295	0.307	1.442	0.260	*p* > 0.05
	TS	166	0.006	0.187	0.807	0.099	0.901	0.187	0.178	1.217	0.162	*p* > 0.05
	HS	201	0.040	0.279	0.682	0.179	0.821	0.279	0.294	1.416	0.251	*p* > 0.05

Note: Ho represents observed heterozygosity, He represents expected heterozygosity, Ne represents the effective number of alleles, and PIC represents polymorphism information content.

**Table 3:** Polymorphism information of InDel locus of sheep MST2 gene.

Locus	Breed	Number	Genotypic Frequency			Allelic Frequency		Polymorphism Information				
		n	II	ID	DD	I	D	Ho	He	Ne	PIC	$x^{2}$
MST2-S1	LLTS	61	0.098	0.377	0.525	0.287	0.713	0.377	0.409	1.693	0.326	*p* > 0.05
	STHS	190	0.079	0.368	0.553	0.263	0.737	0.368	0.388	1.633	0.313	*p* > 0.05
	TS	166	0.042	0.331	0.627	0.208	0.792	0.331	0.329	1.491	0.275	*p* > 0.05
	HS	201	0.139	0.458	0.403	0.368	0.632	0.458	0.465	1.870	0.357	*p* > 0.05
MST2-S4	LLTS	61	0.230	0.541	0.230	0.500	0.500	0.541	0.500	2.000	0.375	*p* > 0.05
	STHS	190	0.311	0.474	0.216	0.547	0.453	0.474	0.496	1.982	0.373	*p* > 0.05
	TS	166	0.211	0.458	0.331	0.440	0.560	0.458	0.493	1.972	0.371	*p* > 0.05
	HS	201	0.294	0.303	0.403	0.445	0.555	0.303	0.494	1.976	0.372	*p* > 0.05
MST2-S15	LLTS	61	0.115	0.459	0.426	0.344	0.656	0.459	0.451	1.823	0.349	*p* > 0.05
MST2-S27	LLTS	61	0.000	0.230	0.770	0.115	0.885	0.230	0.204	1.256	0.183	*p* > 0.05
	STHS	190	0.016	0.142	0.842	0.087	0.913	0.142	0.159	1.189	0.146	*p* > 0.05
	TS	166	0.000	0.289	0.711	0.145	0.855	0.289	0.248	1.330	0.217	*p* > 0.05
	HS	201	0.005	0.214	0.781	0.112	0.888	0.214	0.199	1.248	0.179	*p* > 0.05
MST2-S36	STHS	190	0.721	0.242	0.037	0.842	0.158	0.242	0.266	1.363	0.231	*p* > 0.05
	TS	166	0.500	0.410	0.090	0.705	0.295	0.410	0.416	1.712	0.329	*p* > 0.05
	HS	201	0.547	0.378	0.075	0.736	0.264	0.378	0.389	1.636	0.313	*p* > 0.05
MST2-S38	LLTS	61	0.770	0.180	0.049	0.861	0.139	0.180	0.239	1.315	0.211	*p* > 0.05
	STHS	190	0.626	0.305	0.068	0.779	0.221	0.305	0.344	1.525	0.285	*p* > 0.05
	TS	166	0.494	0.373	0.133	0.681	0.319	0.373	0.434	1.768	0.340	*p* > 0.05
	HS	201	0.537	0.254	0.209	0.664	0.336	0.254	0.446	1.806	0.347	*p* > 0.05
MST2-S40	STHS	190	0.621	0.326	0.053	0.784	0.216	0.326	0.339	1.512	0.281	*p* > 0.05
	TS	166	0.452	0.446	0.102	0.675	0.325	0.446	0.439	1.782	0.342	*p* > 0.05
	HS	201	0.488	0.418	0.095	0.697	0.303	0.418	0.422	1.731	0.333	*p* > 0.05

Note: Ho represents observed heterozygosity, He represents expected heterozygosity, Ne represents the effective number of alleles, and PIC represents polymorphism information content.

**Table 4:** Effects of *MST1* InDel loci on growth traits in sheep.

Locus	Breed	Growth Trait	Observed Genotypes (MEANS ± SEM)			*p*
			II	ID	DD	
MST1-S2	HS	BoH (cm)	62.53 ± 0.39 ^a^ (*n* = 96)	61.04 ± 0.38 ^b^ (*n* = 78)	61.50 ± 0.73 ^ab^ (*n* = 27)	0.027
	HS	BOL (cm)	71.35 ± 0.39 ^a^ (*n* = 96)	70.90 ± 0.46 ^a^ (*n* = 78)	69.04 ± 0.58 ^b^ (*n* = 27)	0.022
MST1-S8	TS	MFoW (cm)	12.91 ± 0.12 ^a^ (*n* = 88)	12.85 ± 0.14 ^a^ (*n* = 64)	12.07 ± 0.13 ^b^ (*n* = 14)	0.026
MST1-S17	TS	BoL (cm)	-	67.71 ± 1.66 ^b^ (*n* = 31)	70.30 ± 0.45 ^a^ (*n* = 134)	0.045
	TS	HeD (cm)	-	14.25 ± 0.25 ^b^ (*n* = 31)	14.76 ± 0.09 ^a^ (*n* = 134)	0.043
	HS	CaC(cm)	6.25 ± 0.27 ^b^ (*n* = 8)	7.13 ± 0.08 ^a^ (*n* = 56)	7.15 ± 0.04 ^a^ (*n* = 137)	0.000
	HS	BW (kg)	28.35 ± 1.26 ^b^ (*n* = 8)	32.72 ± 0.58 ^a^ (*n* = 56)	32.38 ± 0.39 ^a^ (*n* = 137)	0.037

Note: BoH represents body height, BOL represents body oblique length, MFoW represents maximum forehead width, BoL represents body length, HeD represents head depth, CaC represents cannon circumference, and BW represents body weight. If two groups share the same superscript letter, it indicates that there is no significant difference between them (*p* > 0.05). If two groups do not share the same superscript letter, it indicates that there is a significant difference between them (*p* < 0.05).

**Table 5:** Effects of *MST2* InDel loci on growth traits in sheep.

Locus	Breed	Growth Trait	Observed Genotypes (MEANS ± SEM)			*p*
			II	ID	DD	
MST2-S1	HS	BW (kg)	34.34 ± 0.88 ^a^ (*n* = 28)	32.33 ± 0.46 ^b^ (*n* = 92)	31.59 ± 0.50 ^b^ (*n* = 81)	0.021
	STHS	Hih (cm)	63.45 ± 1.24 ^a^ (*n* = 15)	61.82 ± 0.50 ^b^ (*n* = 70)	63.53 ± 0.40 ^a^ (*n* = 105)	0.029
MST2-S4	LLTS	BoL (cm)	68.58 ± 2.18 ^b^ (*n* = 14)	75.77 ± 1.35 ^a^ (*n* = 33)	79.83 ± 3.65 ^a^ (*n* = 14)	0.026
	LLTS	BoH (cm)	70.00 ± 1.86 ^b^ (*n* = 14)	77.10 ± 1.28 ^a^ (*n* = 33)	80.17 ± 2.73 ^a^ (*n* = 14)	0.021
	LLTS	Hih (cm)	74.50 ± 1.61 ^b^ (*n* = 14)	77.83 ± 1.43 ^b^ (*n* = 33)	98.00 ± 17.07 ^a^ (*n* = 14)	0.031
	STHS	CaC (cm)	7.16 ± 0.10 ^a^ (*n* = 59)	7.21 ± 0.07 ^a^ (*n* = 90)	6.79 ± 0.12 ^b^ (*n* = 41)	0.008
MST2-S15	LLTS	BW(kg)	32.50 ± 1.72 ^b^ (*n* = 7)	60.71 ± 3.21 ^a^ (*n* = 28)	52.53 ± 4.09 ^a^ (*n* = 26)	0.017
	LLTS	ChC (cm)	79.00 ± 8.02 ^b^ (*n* = 7)	98.25 ± 1.61 ^a^ (*n* = 28)	94.68 ± 2.55 ^a^ (*n* = 26)	0.012
MST2-S36	HS	CaC (cm)	7.19 ± 0.05 ^a^ (*n* = 110)	6.99 ± 0.07 ^b^ (*n* = 76)	7.10 ± 0.10 ^a b^ (*n* = 15)	0.044
	STHS	BoH (cm)	63.76 ± 0.36 ^a^ (*n* = 137)	62.27 ± 0.58 ^b^ (*n* = 46)	60.39 ± 1.88 ^b^ (*n* = 7)	0.022
	TS	CaC (cm)	7.12 ± 0.11 ^b^ (*n* = 83)	7.42 ± 0.11 ^a b^ (*n* = 68)	7.69 ± 0.30 ^a^ (*n* = 15)	0.039

Note: BW represents body weight, Hih represents hip height, BoL represents body length, BoH represents body height, CaC represents cannon circumference, and ChC represents chest circumference. If two groups share the same superscript letter, it indicates that there is no significant difference between them (*p* > 0.05). If two groups do not share the same superscript letter, it indicates that there is a significant difference between them (*p* < 0.05).

**Table 6:** Polymorphism information of InDel locus of *YAP* and *TAZ* genes in sheep.

Locus	Breed	Number	Genotypic Frequency			Allelic Frequency		Population Parameters				
		n	II	ID	DD	I	D	Ho	He	Ne	PIC	HWE
YAP-S4	LLTS	61	0.016	0.246	0.738	0.139	0.861	0.246	0.239	1.315	0.211	*p* > 0.05
	STHS	190	0.042	0.321	0.637	0.203	0.797	0.321	0.324	1.478	0.271	*p* > 0.05
	HS	201	0.040	0.303	0.657	0.192	0.808	0.303	0.310	1.450	0.262	*p* > 0.05
YAP-S5	LLTS	61	0.066	0.393	0.541	0.262	0.738	0.393	0.387	1.631	0.312	*p* > 0.05
	STHS	190	0.042	0.342	0.616	0.213	0.787	0.342	0.335	1.504	0.279	*p* > 0.05
	HS	201	0.090	0.313	0.597	0.246	0.754	0.313	0.371	1.590	0.302	*p* > 0.05
YAP-S7	LLTS	61	0.738	0.246	0.016	0.861	0.139	0.246	0.239	1.315	0.211	*p* > 0.05
	STHS	190	0.532	0.395	0.074	0.729	0.271	0.395	0.395	1.653	0.317	*p* > 0.05
	HS	201	0.647	0.308	0.045	0.801	0.199	0.308	0.319	1.468	0.268	*p* > 0.05
YAP-S11	LLTS	61	0.705	0.279	0.016	0.844	0.156	0.279	0.263	1.357	0.229	*p* > 0.05
	HS	201	0.955	0.045	0.000	0.978	0.022	0.045	0.043	1.045	0.042	*p* > 0.05
YAP-S15	LLTS	61	0.656	0.328	0.016	0.820	0.180	0.328	0.295	1.419	0.252	*p* > 0.05
	STHS	190	0.763	0.195	0.042	0.861	0.139	0.195	0.239	1.315	0.211	*p* > 0.05
YAP-S17	LLTS	61	0.541	0.410	0.049	0.746	0.254	0.410	0.379	1.610	0.307	*p* > 0.05
	STHS	190	0.295	0.516	0.189	0.553	0.447	0.516	0.494	1.978	0.372	*p* > 0.05
	TS	166	0.795	0.181	0.024	0.886	0.114	0.181	0.202	1.253	0.182	*p* > 0.05
	HS	201	0.393	0.438	0.169	0.612	0.388	0.438	0.475	1.904	0.362	*p* > 0.05
YAP-S19	LLTS	61	0.803	0.197	0.000	0.902	0.098	0.197	0.177	1.215	0.161	*p* > 0.05
	STHS	190	0.895	0.100	0.005	0.945	0.055	0.100	0.104	1.116	0.099	*p* > 0.05
	TS	166	0.693	0.277	0.030	0.831	0.169	0.277	0.281	1.391	0.241	*p* > 0.05
	HS	201	0.776	0.199	0.025	0.876	0.124	0.199	0.217	1.278	0.194	*p* > 0.05
YAP-S21	LLTS	61	0.607	0.344	0.049	0.779	0.221	0.344	0.344	1.525	0.285	*p* > 0.05
	STHS	190	0.558	0.395	0.047	0.755	0.245	0.395	0.370	1.587	0.302	*p* > 0.05
	TS	166	0.614	0.349	0.036	0.789	0.211	0.349	0.333	1.499	0.278	*p* > 0.05
	HS	201	0.791	0.209	0.000	0.896	0.104	0.209	0.186	1.229	0.169	*p* > 0.05
TAZ-S17	LLTS	61	0.311	0.443	0.246	0.533	0.467	0.443	0.498	1.991	0.374	*p* > 0.05
	TS	166	0.301	0.428	0.271	0.515	0.485	0.428	0.500	1.998	0.375	*p* > 0.05
TAZ-S24	TS	166	0.639	0.331	0.030	0.804	0.196	0.331	0.315	1.460	0.266	*p* > 0.05

Note: Ho represents observed heterozygosity, He represents expected heterozygosity, Ne represents the effective number of alleles, and PIC represents polymorphism information content.

**Table 7:** Effects of InDel locus of *YAP* gene on growth traits in sheep.

Locus	Breed	Growth Trait	Observed Genotypes (MEANS ± SEM)			*p*
			II	ID	DD	
YAP-S19	LLTS	BoH (cm)	75.43 ± 1.07 ^b^ (*n* = 49)	85.25 ± 2.85 ^a^ (*n* = 12)	-	0.005
	LLTS	ChD (cm)	34.95 ± 0.59 ^b^ (*n* = 49)	38.88 ± 1.16 ^a^ (*n* = 12)	-	0.035
	TS	BoL(cm)	69.67 ± 0.53 ^b^ (*n* = 115)	69.65 ± 0.92 ^b^ (*n* = 46)	76.00 ± 1.53 ^a^ (*n* = 5)	0.032
YAP-S21	LLTS	ChC (cm)	97.35 ± 2.32 ^a^ (*n* = 37)	94.70 ± 2.01 ^a^ (*n* = 21)	80.17 ± 9.20 ^b^ (*n* = 3)	0.028
	STHS	ChC (cm)	72.58 ± 0.52 ^a^ (*n* = 106)	72.04 ± 0.78 ^a^ (*n* = 75)	66.58 ± 1.38 ^b^ (*n* = 9)	0.015
	TS	RuH (cm)	49.28 ± 0.35 ^b^ (*n* = 102)	50.80± 0.50 ^a^ (*n* = 58)	49.67 ± 0.67 ^ab^ (*n* = 6)	0.047
	TS	HeL (cm)	19.88 ± 0.18 ^b^ (*n* = 102)	20.86 ± 0.22 ^a^ (*n* = 58)	19.50 ± 0.87 ^ab^ (*n* = 6)	0.004
	TS	CaC (cm)	7.11 ± 0.09 ^b^ (*n* = 102)	7.60 ± 0.13 ^a^ (*n* = 58)	7.93 ± 0.35 ^a^ (*n* = 6)	0.004

Note: BoH represents body height, ChD represents chest depth, BoL represents body length, RuH represents rump height, HeL represents head length, ChC represents chest circumference, and CaC represents cannon circumference. If two groups share the same superscript letter, it indicates that there is no significant difference between them (*p* > 0.05). If two groups do not share the same superscript letter, it indicates that there is a significant difference between them (*p* < 0.05).

**Table 8:** Polymorphism information of *MOB1A* gene InDel locus in sheep.

Locus	Breed	Number	Genotypic Frequency			Allelic Frequency		Population Parameters				
		n	II	ID	DD	I	D	Ho	He	Ne	PIC	HWE
MOB1A -S4	LLTS	61	0.754	0.230	0.016	0.869	0.131	0.230	0.228	1.295	0.202	*p* > 0.05
	STHS	190	0.826	0.158	0.016	0.905	0.095	0.158	0.172	1.208	0.157	*p* > 0.05
MOB1A -S7	LLTS	61	0.623	0.361	0.016	0.803	0.197	0.361	0.316	1.463	0.266	*p* > 0.05
	STHS	190	0.505	0.411	0.084	0.711	0.289	0.411	0.411	1.698	0.327	*p* > 0.05
	TS	166	0.530	0.361	0.108	0.711	0.289	0.361	0.411	1.698	0.327	*p* > 0.05
	HS	201	0.438	0.433	0.129	0.654	0.346	0.433	0.453	1.827	0.350	*p* > 0.05
MOB1A -S10	LLTS	61	0.738	0.213	0.049	0.844	0.156	0.213	0.263	1.357	0.229	*p* > 0.05
	STHS	190	0.811	0.137	0.053	0.879	0.121	0.137	0.213	1.270	0.190	*p* > 0.05
	HS	201	0.821	0.144	0.035	0.893	0.107	0.144	0.191	1.236	0.173	*p* > 0.05

Note: Ho represents observed heterozygosity, He represents expected heterozygosity, Ne represents the effective number of alleles, and PIC represents polymorphism information content.

**Table 9:** Effects of InDel locus of *MOB1A* gene on growth traits in sheep.

Locus	Breed	Growth Trait	Observed Genotypes (MEANS ± SEM)			*p*
			II	ID	DD	
MOB1A-S4	STHS	CaC (cm)	7.11 ± 0.06 ^b^ (*n* = 157)	6.95 ± 0.14 ^b^ (*n* = 30)	8.13 ± 0.33 ^a^ (*n* = 3)	0.027
MOB1A-S7	HS	CaC (cm)	6.96 ± 0.06 ^b^ (*n* = 88)	7.20 ± 0.06 ^a^ (*n* = 87)	7.31 ± 0.10 ^a^ (*n* = 26)	0.002
	HS	BW (kg)	31.41 ± 0.49 ^b^ (*n* = 88)	32.86 ± 0.48 ^a^ (*n* = 87)	33.55 ± 0.84 ^a^ (*n* = 26)	0.035
	TS	HiW (cm)	17.52 ± 0.38 ^ab^ (*n* = 88)	16.82 ± 0.36 ^b^ (*n* = 60)	19.00 ± 0.57 ^a^ (*n* = 18)	0.028
	TS	HeL(cm)	20.34 ± 0.19 ^a^ (*n* = 88)	19.73 ± 0.27 ^b^ (*n* = 60)	20.75 ± 0.26 ^a^ (*n* = 18)	0.046
MOB1A-S10	LLTS	CaC (cm)	7.87 ± 0.17 ^a^ (*n* = 45)	6.79 ± 0.32 ^b^ (*n* = 13)	7.25 ± 0.25 ^ab^ (*n* = 3)	0.024
	STHS	ChC (cm)	71.55 ± 0.46 ^b^ (*n* = 154)	74.32 ± 0.49 ^a^ (*n* = 26)	74.50 ± 0.39 ^a^ (*n* = 110)	0.039
	HS	CaC (cm)	7.05 ± 0.04 ^b^ (*n* = 165)	7.31 ± 0.09 ^a^ (*n* = 29)	7.50 ± 0.19 ^a^ (*n* = 7)	0.011

Note: CaC represents cannon circumference, BW represents body weight, HiW represents hip width, HeL represents head length, and ChC represents chest circumference. If two groups share the same superscript letter, it indicates that there is no significant difference between them (*p* > 0.05). If two groups do not share the same superscript letter, it indicates that there is a significant difference between them (*p* < 0.05).

## Data Availability

Data are contained within this manuscript and Supplementary Materials.

## Supplementary Materials

The following supporting information can be downloaded at: https://www.mdpi.com/article/10.3390/vetsci12030283/s1, Supplementary Figure S1: InDel locus map of MST1 and MST2 genes in sheep; Supplementary Figure S2: Sheep YAP and TAZ gene InDel locus map. Supplementary Figure S3: Sheep YAP and TAZ genes InDel locus map. Supplementary Figure S4: InDel locus map of SAV1, LATS1 and LATS2 genes in sheep.

## Abbreviations

The following abbreviations are used in this manuscript:

MDPI	Multidisciplinary Digital Publishing Institute
GPCRs	G Protein-Coupled Receptors
He	Expected heterozygosity
Ho	Observed heterozygosity
HWE	Hardy–Weinberg equilibrium
InDel	Insertion/Deletion
*LATS1/2*	Large tumor suppressor 1/2
MAS	Marker-assisted selection
*MOB1A/B*	MOB kinase activator 1A/B
*MST1/2*	Mammalian Sterile 20-like kinases 1/2
Ne	Effective number of alleles
PIC	Polymorphism information content
*SAV1*	Salvador 1
*TAZ*	Transcriptional coactivator with PDZ-binding motif
*YAP*	Yes- associated protein
