# Supplementary material for: Identification and Analysis of InDel Variants in Key Hippo Pathway Genes and Their Association with Growth Traits in Four Chinese Sheep Breeds
Source: Vet Sci. 2025 Mar 18;12(3):283. doi: 10.3390/vetsci12030283 (PMC11946644; doi:10.3390/vetsci12030283)
Supplement: Supplementary file 1 [file vetsci-12-00283-s001.zip › Supplementary material.pdf]

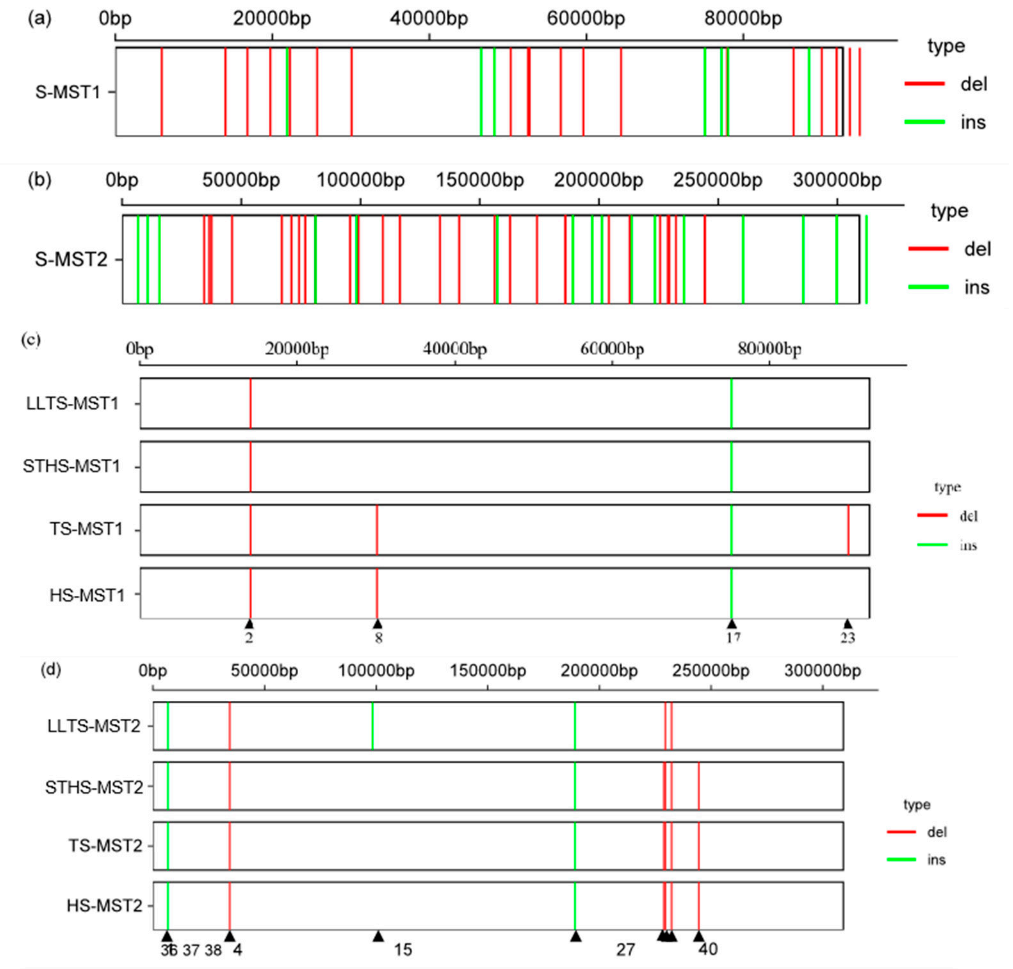


**Supplementary Figure S1** InDel locus map of *MST1* and *MST2* genes in sheep

(a) Schematic diagram of InDel potential sites screened from sheep *MST1* gene ; (b) The InDel potential loci in sheep *MST2* gene ; (c) InDel loci diagram of four sheep *MST1* gene ; (d) InDel loci identified by four sheep *MST2* gene. The red line — del represents the deletion mutation, and the green line — ins represents the insertion mutation.

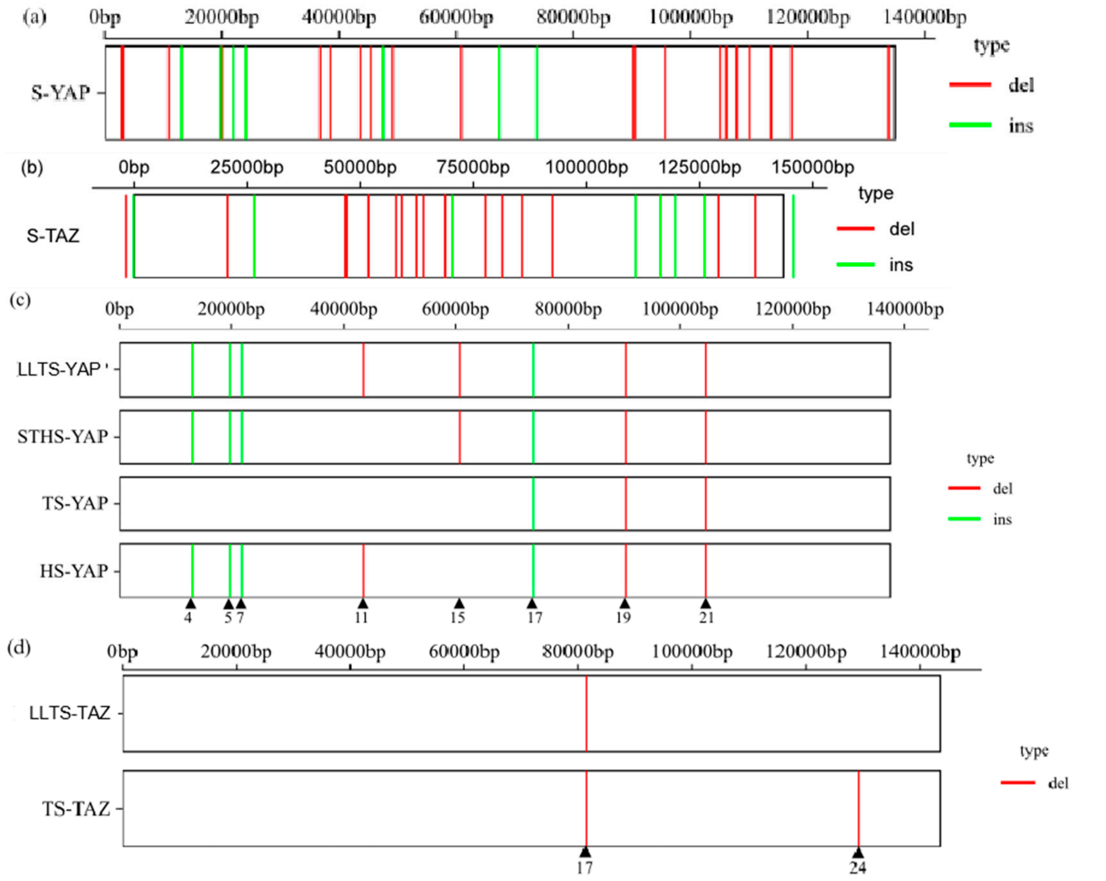


**Supplementary Figure S2** Sheep YAP and TAZ gene InDel locus map.

(a) The schematic diagram of the potential sites of InDel screened from sheep YAP gene ; ( b ) Schematic diagram of InDel potential sites screened from sheep TAZ gene ; ( c ) InDel loci diagram of four sheep YAP genes ; ( d ) InDel locus diagram of TAZ gene identified in Lanzhou large-tailed sheep and Tong sheep. The red line — del represents the deletion mutation, and the green line — ins represents the insertion mutation.

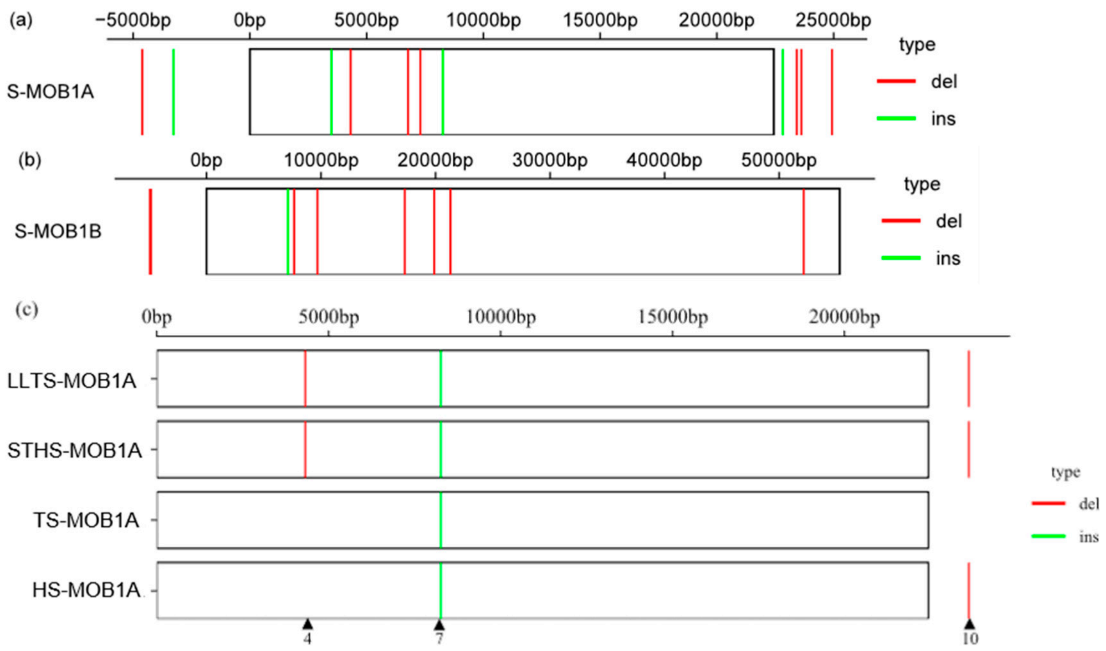


**Supplementary Figure S3** Sheep *YAP* and *TAZ* genes InDel locus map

(a) The schematic diagram of the potential sites of InDel screened from sheep *YAP* gene ; (b) Schematic diagram of InDel potential sites screened from sheep *TAZ* gene ; (c) InDel loci diagram of four sheep *YAP* genes ; (d) InDel locus diagram of *TAZ* gene identified in Lanzhou fat-tailed sheep and Tong sheep. The red line — del represents the deletion mutation, and the green line — ins represents the insertion mutation.

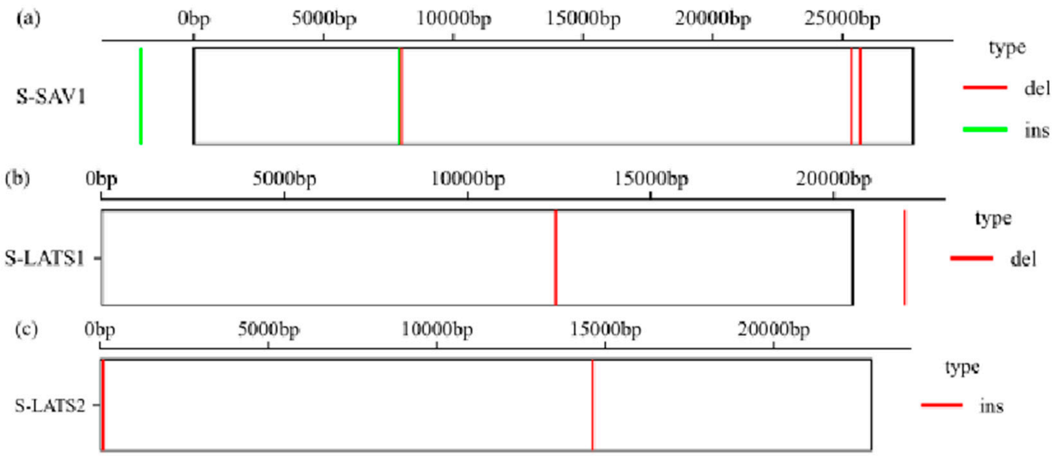


**Supplementary Figure S4** InDel locus map of *SAV1*, *LATS1* and *LATS2* genes in sheep

( a ) The schematic diagram of the potential InDel sites screened from the sheep *SAV1* gene ; ( b ) The schematic diagram of InDel potential sites screened from sheep *LATS1* gene ; ( c ) Schematic diagram of InDel potential sites screened from sheep *LATS2* gene. The red line — del represents the deletion mutation, and the green line — ins represents the insertion mutation.
