# Supplementary figures and images for: Identification and Analysis of InDel Variants in Key Hippo Pathway Genes and Their Association with Growth Traits in Four Chinese Sheep Breeds
Source: Vet Sci. 2025 Mar 18;12(3):283. doi: 10.3390/vetsci12030283 (PMC11946644; doi:10.3390/vetsci12030283)

Original Images for Blots or Gels:

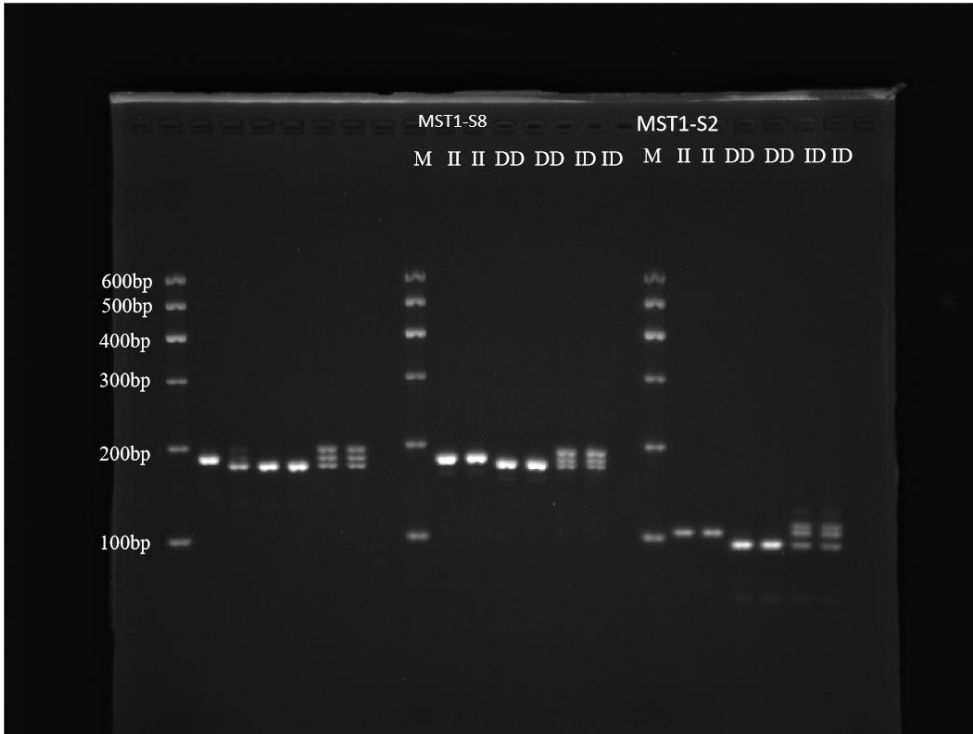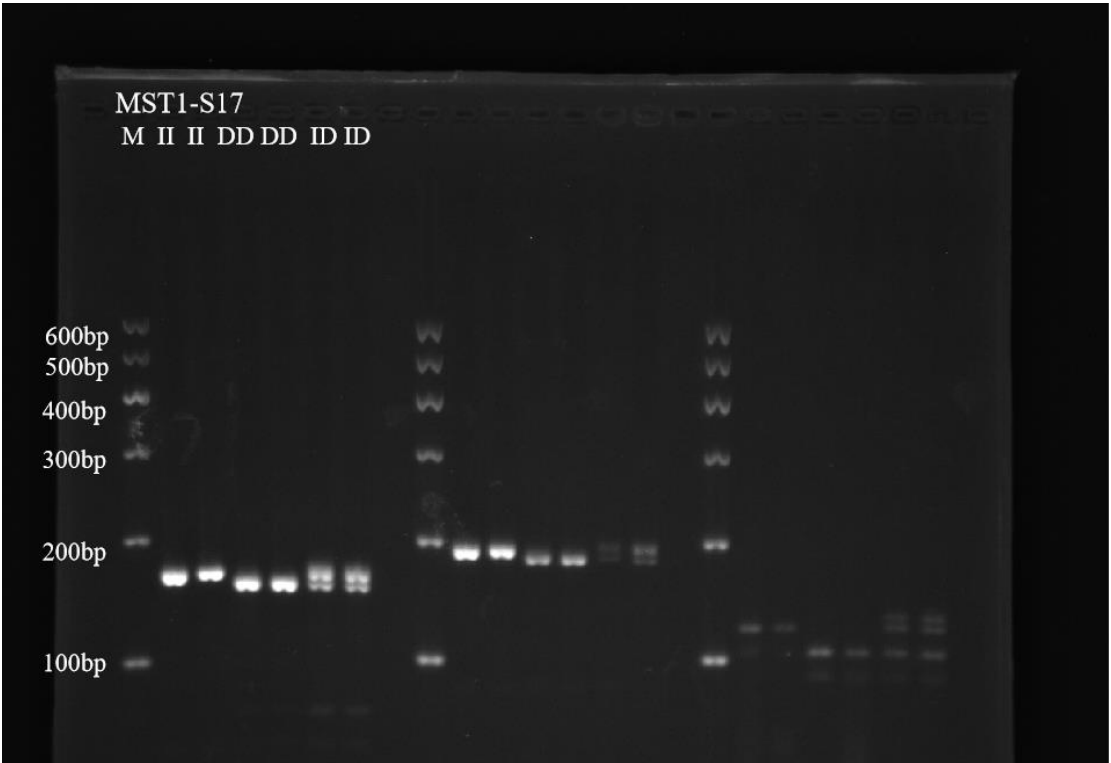

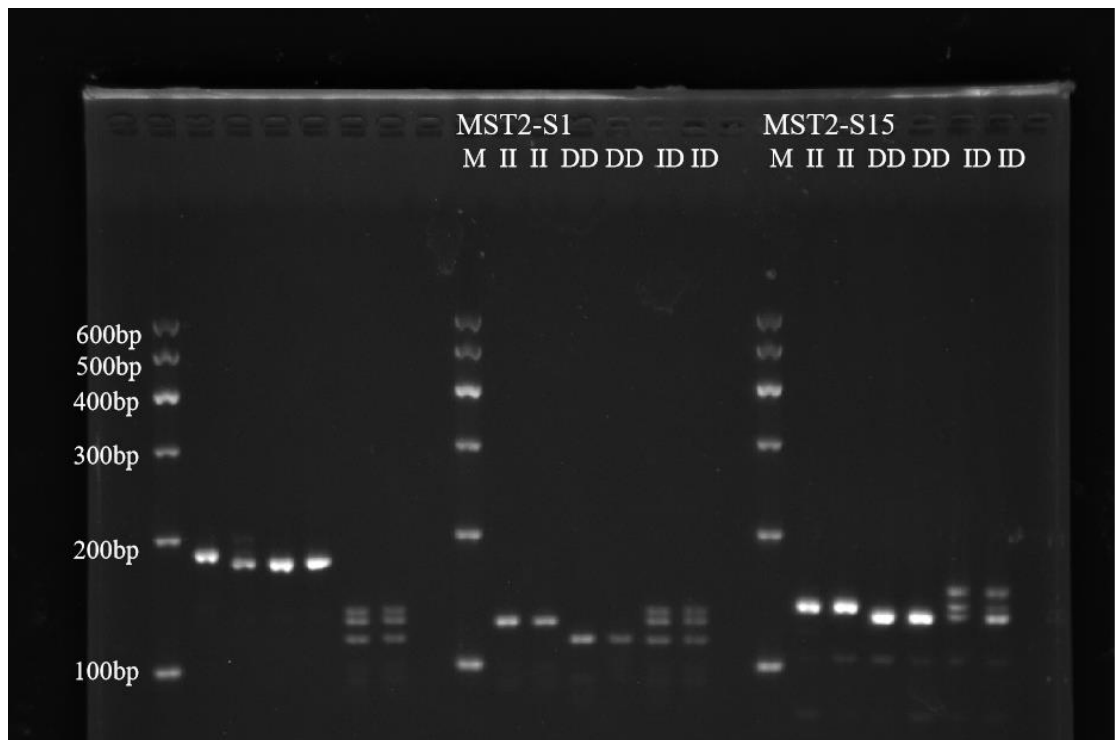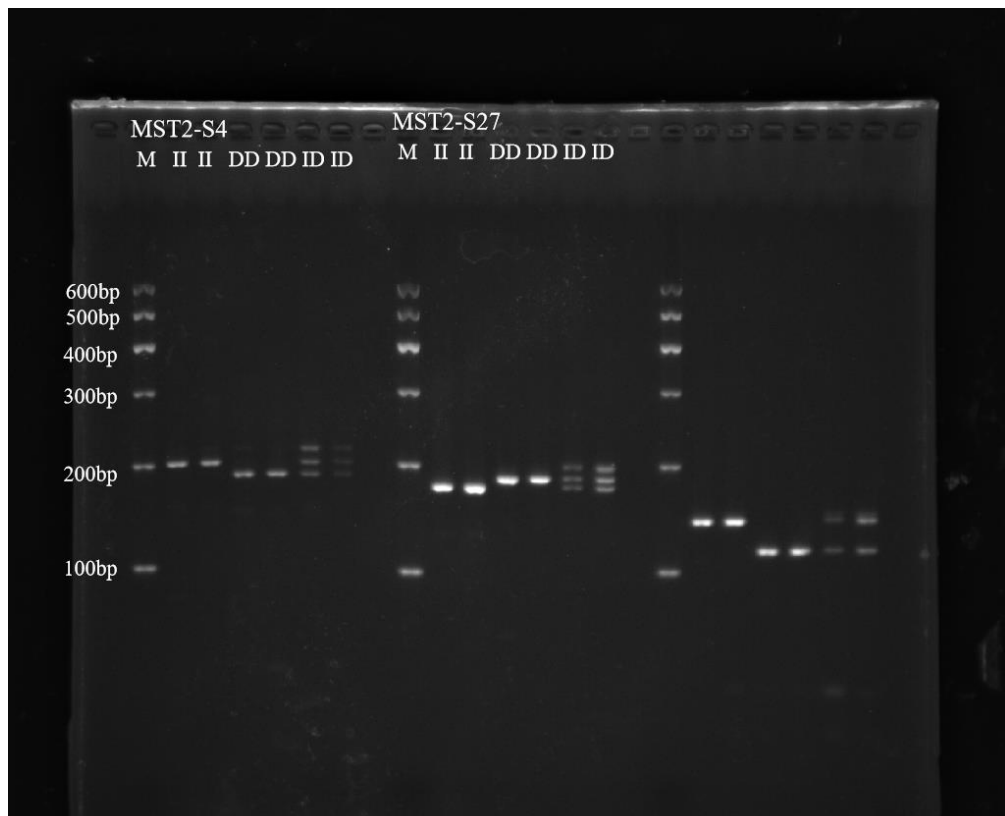

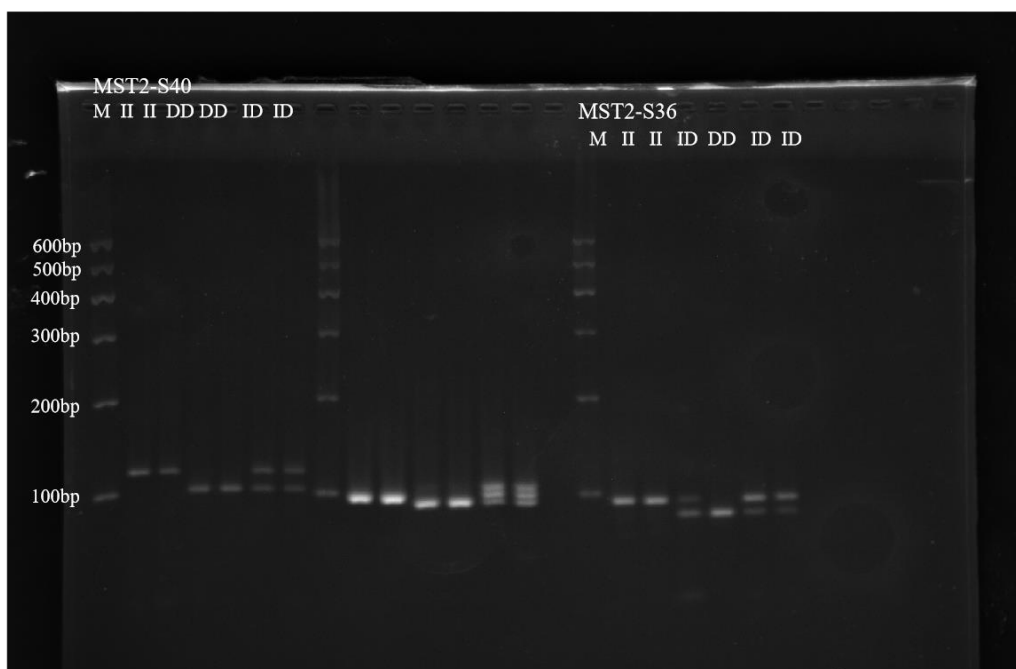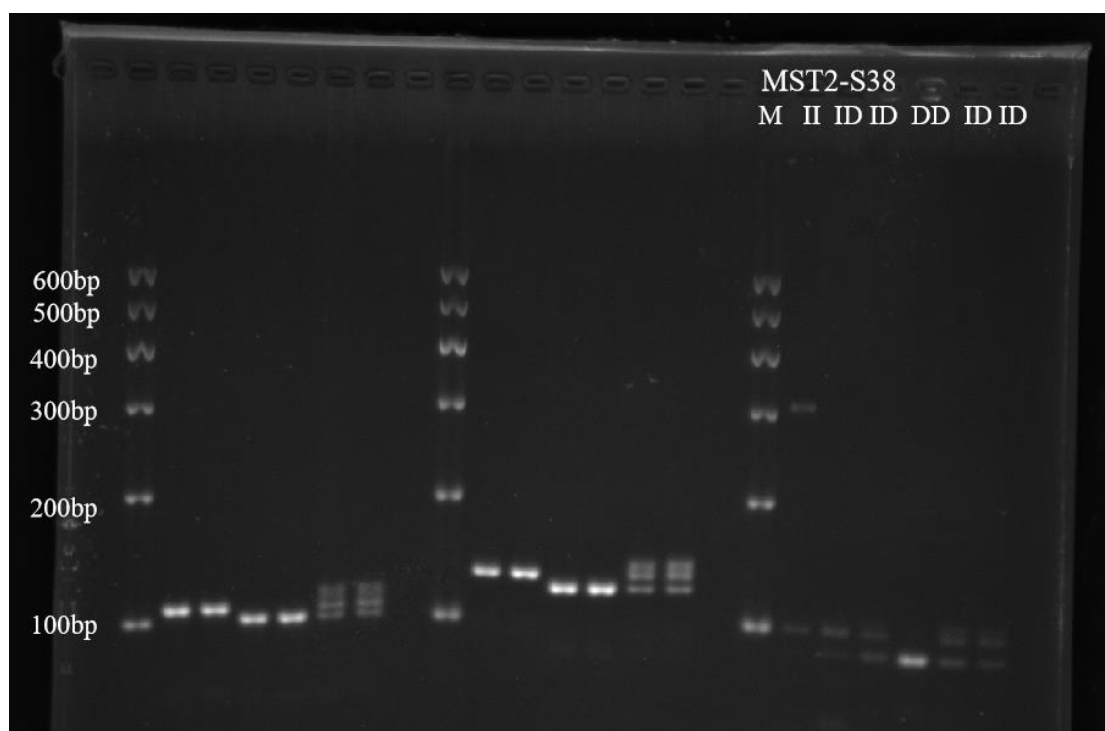

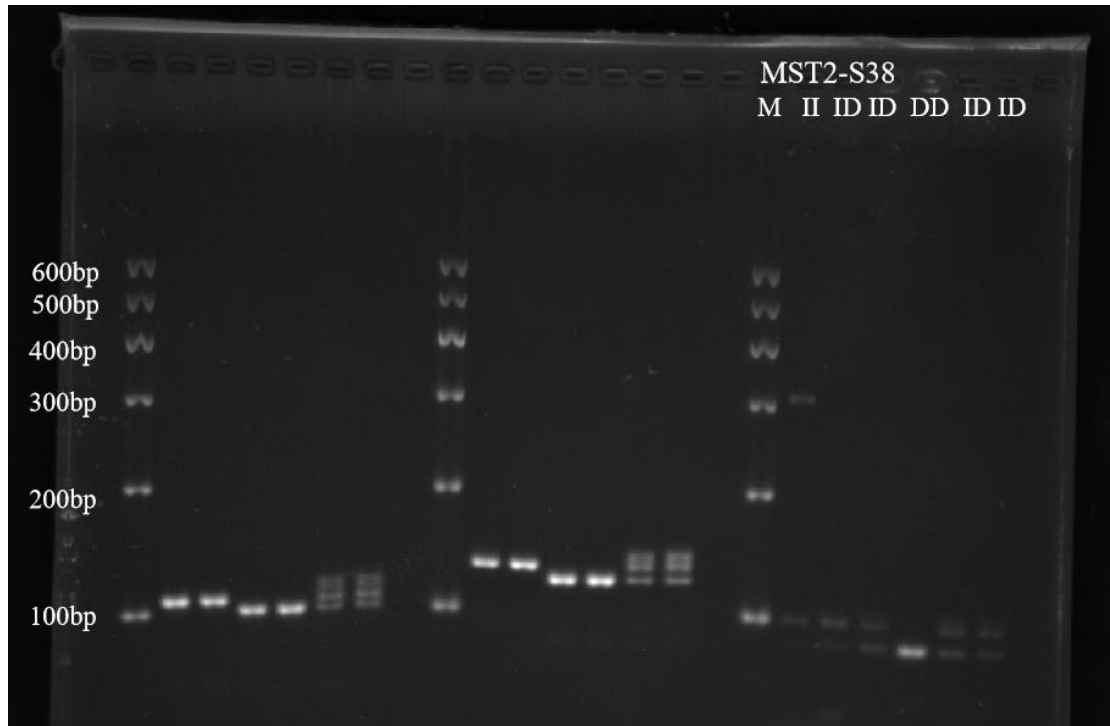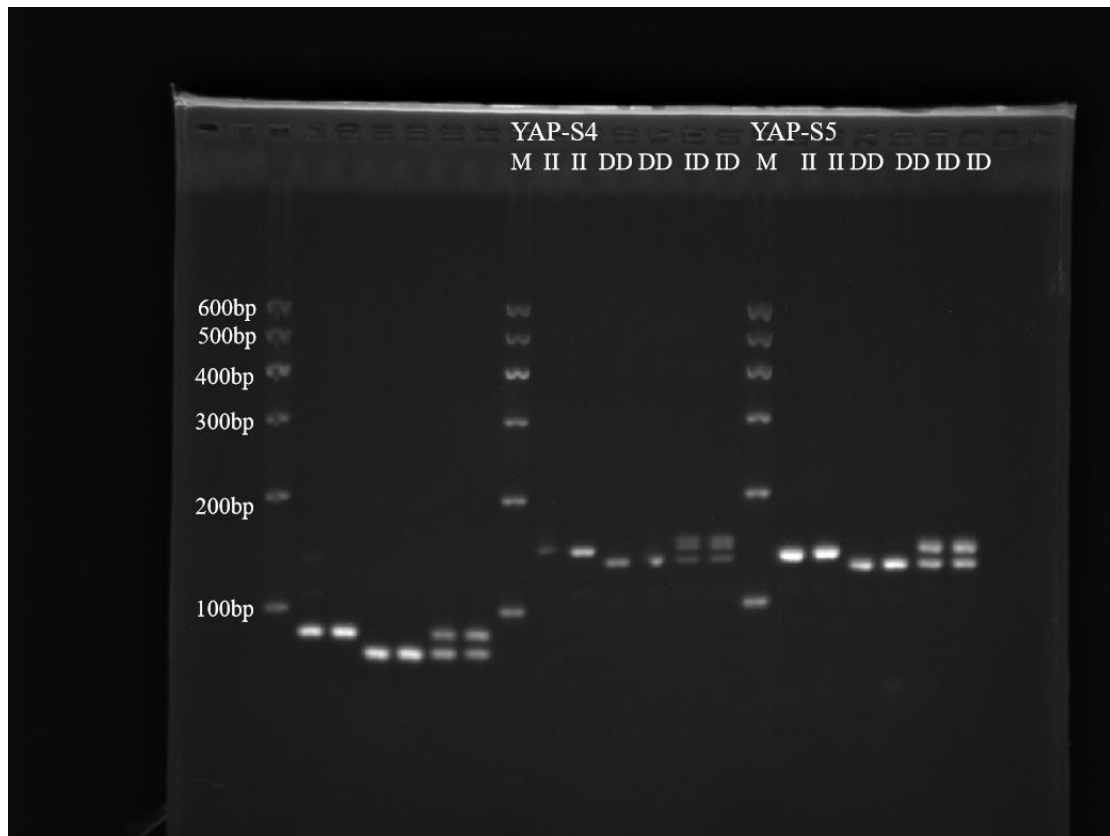

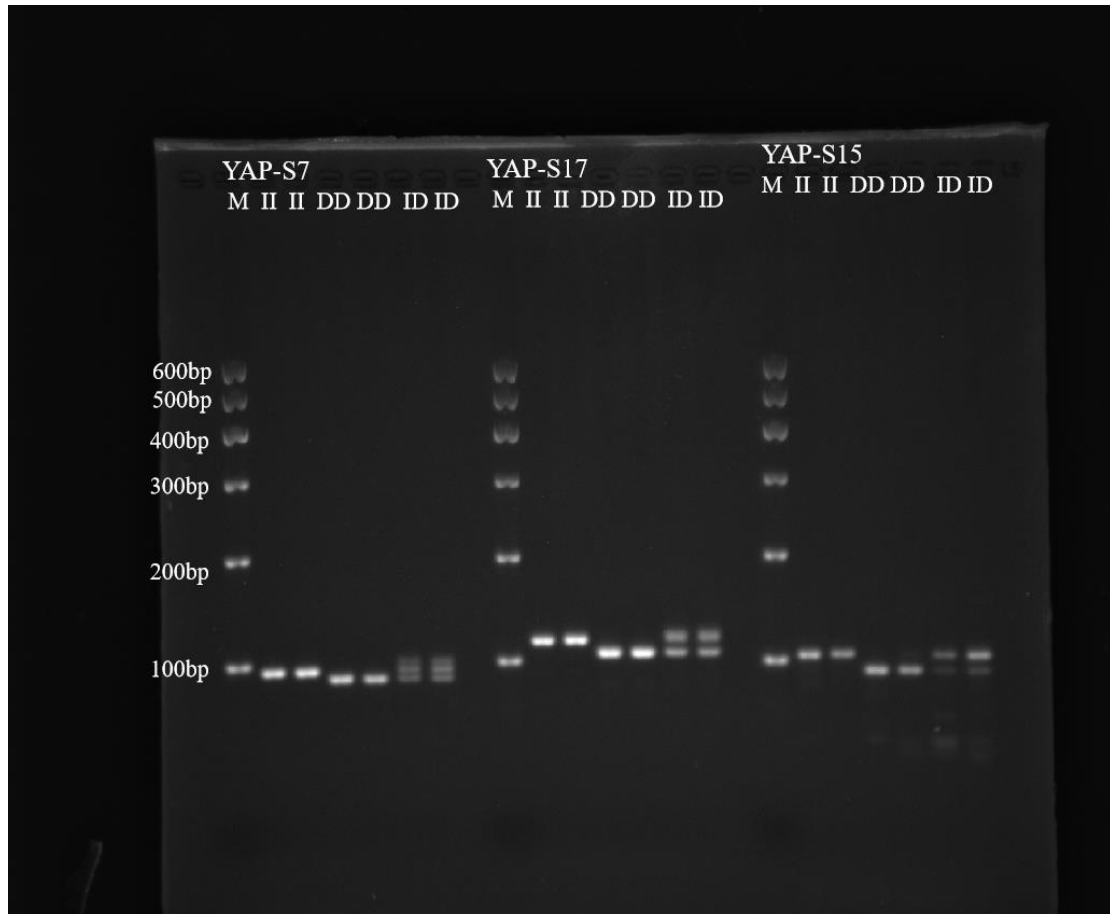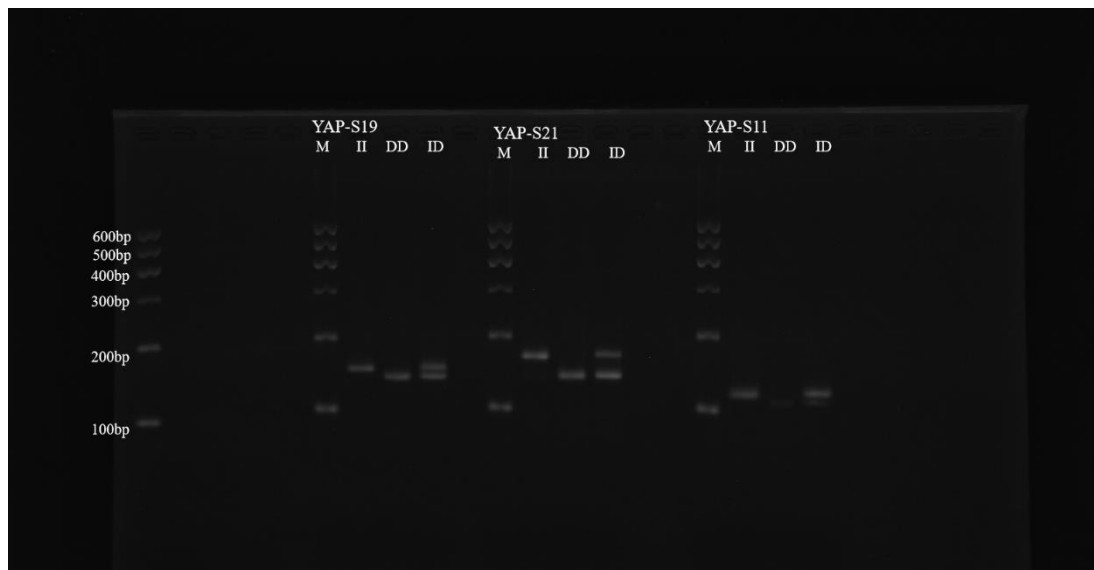

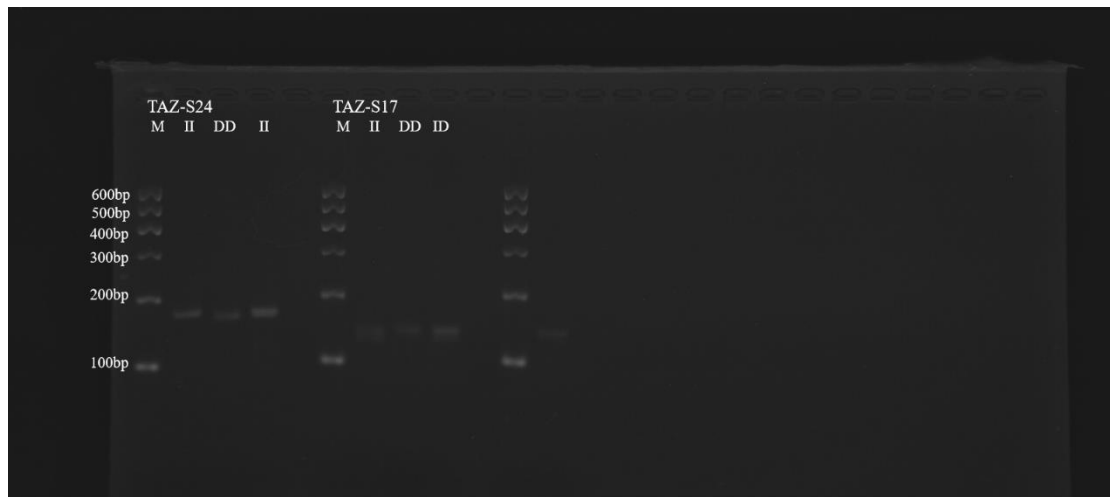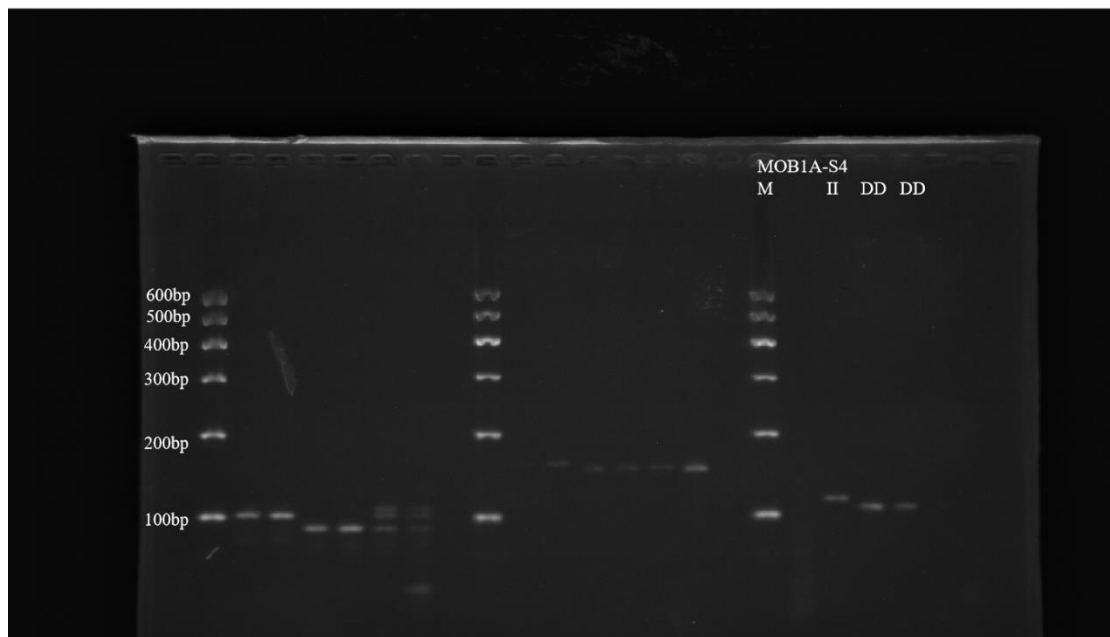

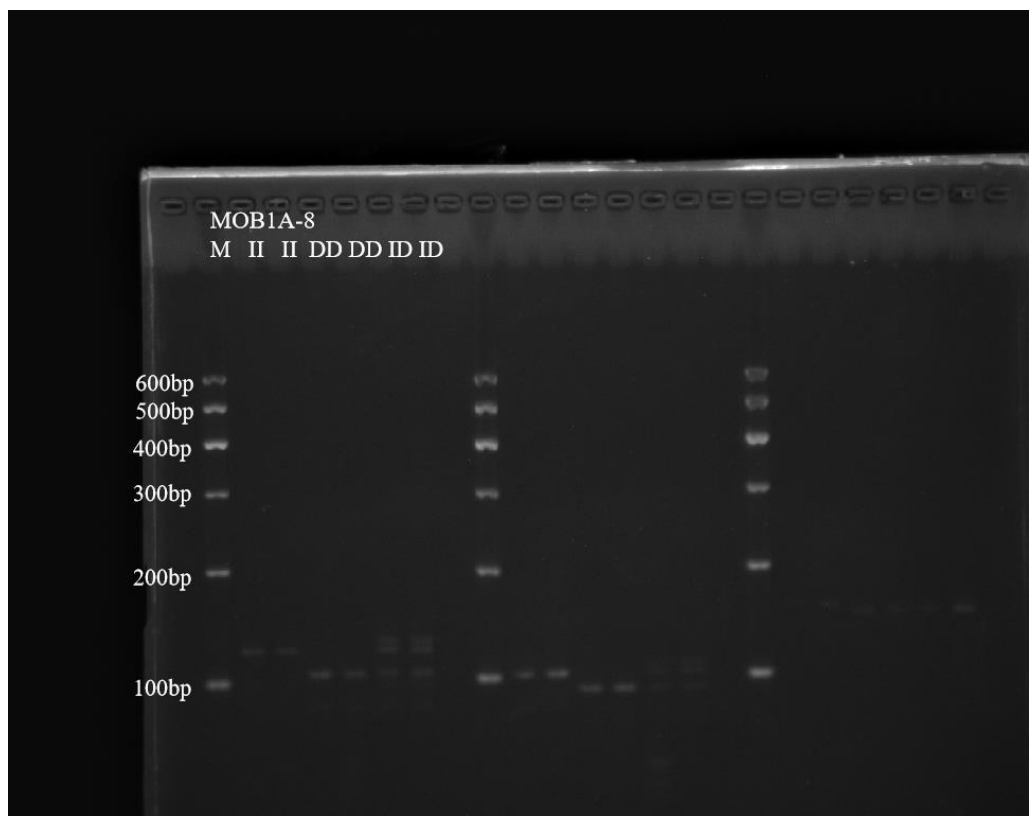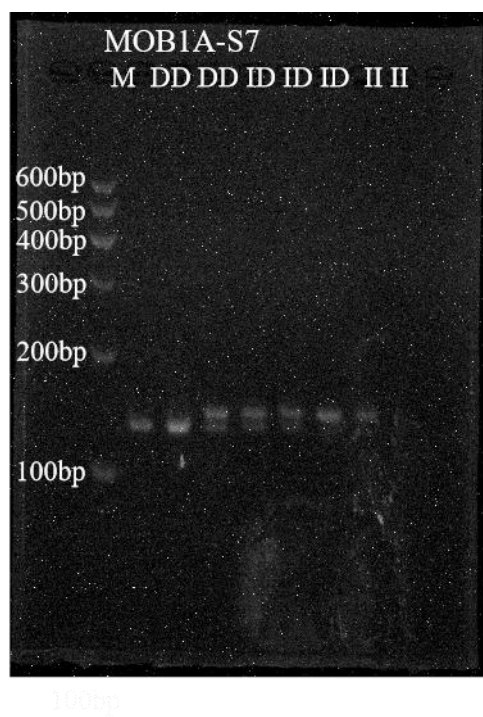

Supplement: Supplementary file 1 [file vetsci-12-00283-s001.zip › vetsci-3446839-WB.pdf]
